# Erector Spinae Plane Block Versus Thoracic Paravertebral Block for Postoperative Analgesia in Thoracic Surgery: A Systematic Review and Meta-Analysis of Randomized and Observational Studies

**DOI:** 10.3390/jcm15041370

**Published:** 2026-02-09

**Authors:** Yoon Ji Choi, Hyun Kang, Sang Hun Kim

**Affiliations:** 1 Department of Anesthesiology and Pain Medicine, Korea University Ansan Hospital, Korea University College of Medicine, Ansan 15355, Republic of Korea; yoonji07@gmail.com; 2 Department of Anesthesiology and Pain Medicine, Chung-Ang University College of Medicine, 84 Heukseok-ro, Dongjak-gu, Seoul 06974, Republic of Korea; roman00@naver.com; 3 Department of Anesthesiology and Pain Medicine, Chosun University Hospital, College of Medicine, Chosun University, 365 Pilmun-daero, Dong-gu, Gwangju 61453, Republic of Korea; 4 Medical Research Institute, Chosun University, 309 Pilmun-daero, Dong-gu, Gwangju 61452, Republic of Korea

**Keywords:** erector spinae plane block, thoracic paravertebral block, thoracic surgery, postoperative analgesia, meta-analysis

## Abstract

**Background/Objectives:** Both erector spinae plane block (ESPB) and thoracic paravertebral block (TPVB) are widely used for thoracic surgery analgesia, but comparative evidence remains inconsistent. This meta-analysis compared their analgesic efficacy and safety with time-stratified analyses and trial sequential analysis (TSA). **Methods:** We searched MEDLINE, Embase, Web of Science, and CENTRAL (inception to January 2026) for randomized controlled trials (RCTs) and observational studies comparing ESPB with TPVB in adults undergoing thoracic surgery. Primary outcomes were pain scores at rest and during coughing at 0–6 h, 24 h, and 48 h postoperatively. Secondary outcomes included opioid consumption and adverse events. Random-effects meta-analyses were performed. Evidence certainty was assessed using GRADE. **Results:** Twenty-five studies (22 RCTs, 3 observational studies; 1847 patients) were included. TPVB provided superior early analgesia (0–6 h) at rest (SMD 0.25, 95% CI 0.03–0.47) and during coughing (SMD 0.28, 95% CI 0.02–0.54); TSA confirmed firm evidence for early pain at rest. Pain scores at 24 h and 48 h were comparable between techniques. TPVB reduced 24 h opioid consumption (SMD 0.42, 95% CI 0.11–0.73), but evidence certainty was low due to heterogeneity and insufficient information size by TSA. No differences were observed in postoperative nausea and vomiting or hypotension. **Conclusions:** ESPB and TPVB provide comparable analgesia beyond the early postoperative period. TPVB demonstrates superior early analgesia (0–6 h) with firm evidence, but opioid-sparing effects remain uncertain. Both techniques are safe. ESPB represents a practical alternative to TPVB, particularly where technical simplicity is prioritized.

## 1. Introduction

Effective analgesic strategies after thoracic surgery contribute to improved recovery trajectories and a lower risk of postoperative complications. Significant postoperative pain frequently follows thoracotomy and video-assisted thoracoscopic surgery (VATS), influencing respiratory performance and functional recovery, and may be associated with the development of long-lasting postoperative pain [1,2]. Inadequate pain control during the early postoperative period can further suppress effective coughing and deep breathing, thereby increasing pulmonary complications [3].

Thoracic paravertebral block (TPVB) is a well-established regional analgesic modality in thoracic surgical practice, with well-established efficacy in reducing pain scores and opioid consumption [4,5]. However, TPVB is technically demanding and carries risks such as pneumothorax, vascular injury, and unintended neuraxial spread, potentially limiting its use in certain patient populations [6].

The erector spinae plane block (ESPB) has emerged as a technically simpler and potentially safer alternative [7]. Performed under ultrasound guidance, ESPB involves injection of local anesthetic deep into the erector spinae muscle, with proposed analgesic mechanisms including diffusion into the paravertebral space or modulation of spinal nerve rami [7,8]. Despite its increasing clinical adoption, the precise mechanism and relative analgesic efficacy of ESPB remain incompletely understood.

To date, randomized controlled trials (RCTs) and observational studies (OSs) comparing ESPB and TPVB have reported inconsistent results, particularly regarding early postoperative analgesia and opioid requirements. These discrepancies may reflect differences in study design, timing of outcome assessment, and perioperative analgesic protocols, contributing to ongoing uncertainty in clinical decision-making.

Although previous meta-analyses have attempted to synthesize available evidence, many were limited by small sample sizes, heterogeneous outcome definitions, lack of time-specific analyses, or restricted evaluation of safety outcomes [9,10]. Therefore, this study presents a systematic synthesis of the available evidence comparing ESPB and TPVB in adult patients undergoing thoracic surgery. By incorporating both RCTs and OSs, applying time-stratified outcome analyses, and using trial sequential analysis, this study aims to provide clinically applicable evidence to support regional analgesic decision-making in thoracic surgery.

## 2. Materials and Methods

### 2.1. Study Design and Registration

This systematic review and meta-analysis followed a prespecified protocol developed prior to data extraction and analysis. The study was planned and reported in alignment with the methodological principles outlined in the PRISMA 2020 statement, with the completed checklist provided in the Supplementary Materials (File S1).

A prespecified protocol was recorded in the International Prospective Register of Systematic Reviews before the review commenced (PROSPERO; registration number CRD420261277015) on 3 January 2026. All eligibility criteria, outcome definitions, and analytical approaches were defined a priori to reduce the risk of selective outcome reporting and analytical bias.

### 2.2. Eligibility Criteria

Eligibility was determined using prespecified criteria covering patient characteristics, interventions, comparators, outcome measures, and study design. The population included adult patients (≥18 years) undergoing thoracic surgery, including thoracotomy and VATS. The intervention of interest was ESPB, administered as either a single-shot or continuous technique, and the comparator was TPVB, performed using single-shot or continuous approaches.

The primary outcomes were postoperative pain scores at rest and during coughing or movement, assessed using VAS or NRS. These outcomes were analyzed both as overall pooled estimates and at predefined postoperative time points (0–6 h, 24 h, and 48 h). Secondary outcomes included cumulative opioid consumption, evaluated overall and at 24 h and 48 h after surgery with conversion to oral morphine equivalents, as well as adverse events such as PONV and hypotension. Eligible study designs included RCTs and OSs, including cohort and case–control studies.

Only peer-reviewed full-text studies involving elective thoracic surgery and reporting at least one primary outcome were included. Multi-arm studies were considered eligible when extractable data for a direct ESPB versus TPVB comparison were available and concomitant analgesic regimens were identical between groups. Studies involving pediatric populations, non-thoracic procedures, non-comparative designs, or insufficient quantitative data were excluded. Given the limited number of RCTs, OSs were additionally included to enhance clinical relevance.

### 2.3. Search Strategy

Literature relevant to comparisons between ESPB and TPVB in thoracic surgery was systematically identified through searches of four electronic databases: MEDLINE (via PubMed), Embase (via Ovid), Web of Science, and CENTRAL. Searches covered the period from database inception to 5 January 2026.

Search queries incorporated controlled vocabulary terms and text-based keywords describing ESPB, TPVB, and thoracic surgical procedures. For each database, search expressions were adapted to reflect database-specific syntax and indexing conventions.

The search strategy was structured around three core concepts: (1) erector spinae plane block, (2) thoracic paravertebral block, and (3) thoracic surgery–related procedures. The full list of search terms, Boolean operators, and database-specific adaptations is provided in File S2.

To identify studies not retrieved through database searches, clinical trial registries were additionally searched using keywords consistent with the primary search concepts. Bibliographies of included articles and relevant systematic reviews were also examined to identify further eligible studies. No restrictions on language or publication date were applied.

### 2.4. Study Selection

All retrieved records were managed using EndNote X9 (Clarivate Analytics, Philadelphia, PA, USA), and duplicate entries were identified and removed. Title and abstract screening were performed independently by two reviewers, after which potentially eligible studies underwent full-text evaluation. Discrepancies were resolved through discussion, and a third reviewer was consulted when agreement could not be achieved. Decisions made during full-text assessment, including reasons for exclusion, were documented. The study selection process is summarized in a PRISMA 2020 flow diagram.

### 2.5. Data Extraction

Data extraction was performed independently by two reviewers using a prespecified standardized form. Information collected comprised bibliographic details (first author, year of publication, country, and study design), the number of participants in each treatment group, and surgical characteristics, including thoracotomy and VATS.

The primary outcomes were postoperative pain scores assessed at rest and during coughing or movement using VAS or NRS. These outcomes were evaluated both as pooled estimates across all reported perioperative time points and at predefined postoperative intervals of 0–6 h, 24 h, and 48 h. When outcome data were not reported at the exact predefined time points, the closest available values were extracted; if multiple measurements were available within the 0–6 h postoperative window, the earliest reported value was selected.

Cumulative opioid consumption was analyzed as an overall pooled estimate and separately at 24 h and 48 h after surgery. All opioid doses were converted to oral morphine equivalent doses (OMEDs) to ensure comparability across studies. Secondary outcomes included adverse events such as postoperative nausea and vomiting (PONV) and hypotension.

In multi-arm studies, data were extracted exclusively from the ESPB and TPVB groups when a direct comparison was available and concomitant analgesic regimens were identical across groups. When continuous outcomes were reported as medians with interquartile ranges, corresponding means and standard deviations were estimated using established statistical conversion methods. When numerical outcome data were presented exclusively in graphical form, values were extracted using WebPlotDigitizer software (version 4.6; Ankit Rohatgi, Austin, TX, USA).

All extracted data were independently verified by both reviewers. Initial disagreements were reconciled through joint evaluation, with an independent third reviewer consulted for remaining differences.

### 2.6. Quality Assessment and Risk of Bias Assessment

Methodological quality and risk of bias were evaluated according to study design by two reviewers working independently. RCTs were assessed using the Cochrane RoB 2 tool (Cochrane Collaboration, London, UK), whereas OSs were evaluated with the Risk of Bias Assessment tool for Non-randomized Studies (RoBANS; National Evidence-based Healthcare Collaborating Agency, Seoul, Republic of Korea) [11,12]. Any differences in judgment were resolved through discussion, with consultation of a third reviewer when required. The outcomes of the risk-of-bias assessments are presented in tabular format.

### 2.7. Statistical Analysis

Statistical analyses were conducted using Comprehensive Meta-Analysis software (version 2.0; Biostat Inc., Englewood, NJ, USA). Continuous outcomes were summarized as standardized mean differences (SMDs) with corresponding 95% confidence intervals (CIs), whereas dichotomous outcomes were expressed as risk ratios with 95% CIs. All analyses were performed using two-sided tests, with statistical significance defined as a *p*-value of less than 0.05.

Between-study heterogeneity was evaluated using Cochran’s Q statistic and the I^2^ metric, with the between-study standard deviation (τ) estimated via the DerSimonian–Laird approach. A random-effects model was specified a priori and applied consistently across all analyses, independent of the observed level of heterogeneity. Prediction intervals were derived when τ^2^ exceeded zero. Prespecified subgroup analyses based on study design and sensitivity analyses using a leave-one-out strategy were conducted to assess the robustness of the pooled estimates.

Assessment of publication bias was conducted for outcomes reported by at least 10 studies. Funnel plots were examined to evaluate potential asymmetry, and small-study effects were assessed using Egger’s regression test and Begg’s rank correlation test. When evidence suggestive of publication bias was identified, trim-and-fill analyses were applied to explore its possible influence on the pooled effect estimates.

### 2.8. Trial Sequential Analysis

Trial sequential analysis (TSA) was undertaken to evaluate whether the accumulated evidence was sufficient to support definitive conclusions for the primary outcomes. TSA was performed using TSA software (version 0.9.5.10 Beta; Copenhagen Trial Unit, Copenhagen, Denmark) and was restricted to data derived from RCTs.

The analysis was designed using a two-tailed significance threshold of 0.05 and an assumed power of 80%. The required information size (RIS) and trial sequential monitoring boundaries for benefit, harm, and futility were calculated for both continuous and dichotomous outcomes. Evidence was considered conclusive when the cumulative Z-curve crossed a trial sequential monitoring boundary or reached the RIS.

### 2.9. Certainty of Evidence

The certainty of evidence for each outcome was assessed using the Grading of Recommendations Assessment, Development and Evaluation (GRADE) approach and rated as high, moderate, low, or very low.

## 3. Results

### 3.1. Study Selection and Characteristics

**Figure 1:**
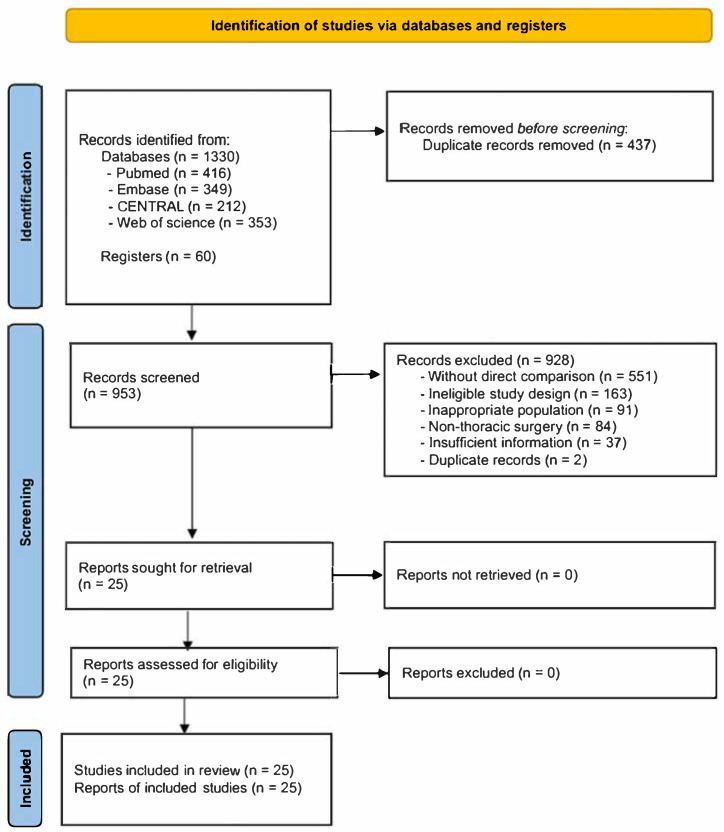
PRISMA 2020 flow diagram summarizing study identification and selection. Database searches yielded 1390 records, from which 437 duplicate entries were removed. Screening of titles and abstracts resulted in the exclusion of 928 records for reasons including unsuitable comparators, non-eligible study designs, irrelevant populations or surgical indications, inadequate information, or duplication. The remaining 25 studies satisfied the inclusion criteria and were incorporated into the qualitative and quantitative analyses.

The literature search yielded 1390 records from electronic databases (PubMed, *n* = 416; Embase, *n* = 349; CENTRAL, *n* = 212; Web of Science, *n* = 353) and an additional 60 records from clinical trial registries. Following the elimination of 437 duplicate entries, 953 records underwent title and abstract screening. Of these, 928 were excluded for lack of relevance to the research objective, leaving 25 articles for full-text evaluation. All 25 articles met the eligibility criteria and were included in the systematic review and meta-analysis (Figure 1).

Overall, 25 studies comprising 1834 patients were included, refs. [13,14,15,16,17,18,19,20,21,22,23,24,25,26,27,28,29,30,31,32,33,34,35,36,37], consisting of 22 RCTs (1423 patients) [13,14,15,16,17,18,19,20,21,22,23,24,25,26,27,28,29,30,31,32,33,34] and three OSs (411 patients) [35,36,37]. Key characteristics of the included studies are presented in Table 1.

### 3.2. Risk of Bias Assessment

The risk of bias for RCTs was evaluated using the Cochrane RoB 2 tool (Table 2). Overall, four trials (16.0%) were assessed as having a low risk of bias across all domains, while the remaining 21 trials (84.0%) were judged to present some concerns, mainly related to deviations from intended interventions and outcome measurement. None of the included RCTs was considered to be at high risk of bias.

The risk of bias in non-randomized studies was assessed using the RoBANS tool (Table 3). All three OSs were considered to have a high overall risk of bias, primarily attributable to confounding, absence of blinding in outcome assessment, and unclear reporting of selective outcomes. Detailed domain-level assessments are presented in Table 2 and Table 3.

### 3.3. Results of the Meta-Analysis

#### 3.3.1. Pain at Rest

##### Overall Pain at Rest Across All Time Points

A total of 25 studies (1834 patients) reported pain at rest [13,14,15,16,17,18,19,20,21,22,23,24,25,26,27,28,29,30,31,32,33,34,35,36,37], including 22 RCTs (1423 patients) [13,14,15,16,17,18,19,20,21,22,23,24,25,26,27,28,29,30,31,32,33,34] and three OSs (411 patients) [35,36,37]. Analysis across all postoperative assessment times up to 72 h showed no overall difference in pain at rest between the ESPB and TPVB groups (SMD, 0.109; 95% CI, −0.037 to 0.255; I^2^ = 55.181; P_chi_^2^ < 0.001; τ = 0.266; 95% PI, −0.441 to 0.659). Stratification by study design demonstrated that, in RCTs, pain at rest was significantly lower with TPVB than with ESPB groups (SMD, 0.137; 95% CI, 0.031 to 0.243; I^2^ = 58.574; P_chi_^2^ < 0.001; τ = 0.303; 95% PI, −0.494 to 0.768). By contrast, analyses of OSs did not show a significant difference between groups (*n* = 3; SMD, −0.058; 95% CI, −0.441 to 0.325; I^2^ = 0.0; P_chi_^2^ = 0.768; τ = 0.0) (Figure S1a, Table S1). Sensitivity analysis showed that the pooled effect estimate varied with the exclusion of individual studies, while the overall direction of the effect remained unchanged (Figure S2a).

##### Early Postoperative Pain at Rest (0–6 h)

**Figure 2:**
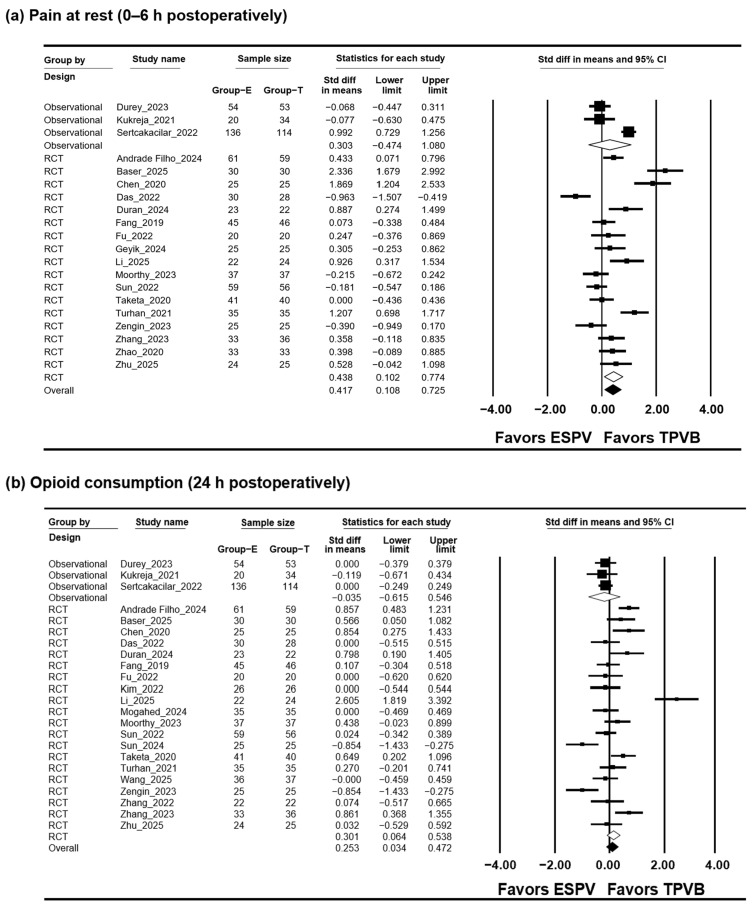
Primary postoperative analgesic outcomes. (**a**) Postoperative pain at rest during the first 0–6 h is presented as SMDs with 95% CIs, where positive values reflect higher pain scores in the ESPB group compared with the TPVB group. (**b**) Cumulative opioid consumption within 24 h is expressed as SMDs with 95% CIs after conversion to oral morphine equivalents; positive values denote greater opioid use in the ESPB group than in the TPVB group [13,14,15,16,17,18,19,20,21,22,23,24,25,26,27,28,29,30,31,32,33,34,35,36,37].

Early postoperative pain at rest was evaluated in 23 studies including 1718 patients [13,14,15,16,17,18,19,21,22,23,24,25,26,27,28,29,30,31,32,34,35,36,37], comprising 20 RCTs (1307 patients) [13,14,15,16,17,18,19,21,22,23,24,25,26,27,28,29,30,31,32,34] and three OSs (411 patients) [35,36,37]. In the overall pooled analysis including both RCTs and OSs, early postoperative pain at rest was lower in the TPVB group than in the ESPB group (SMD, 0.253; 95% CI, 0.034 to 0.472; I^2^ = 79.047; P_chi_^2^ < 0.001; τ = 0.436; 95% PI, −0.651 to 1.157). When analyses were restricted to RCTs, the difference remained statistically significant (SMD, 0.301; 95% CI, 0.064 to 0.538; I^2^ = 80.587; P_chi_^2^ < 0.001; τ = 0.269; 95% PI, −0.262 to 0.864). No significant between-group difference was observed in OSs (SMD, −0.035; 95% CI, −0.615 to 0.546; I^2^ = 0.0; P_chi_^2^ = 0.926; τ = 0.0). (Figure 2a, Table S1). Sensitivity analysis showed that exclusion of the study by Li et al. [22] resulted in the loss of statistical significance without altering the direction of effect (Figure S2b).

**Figure 3:**
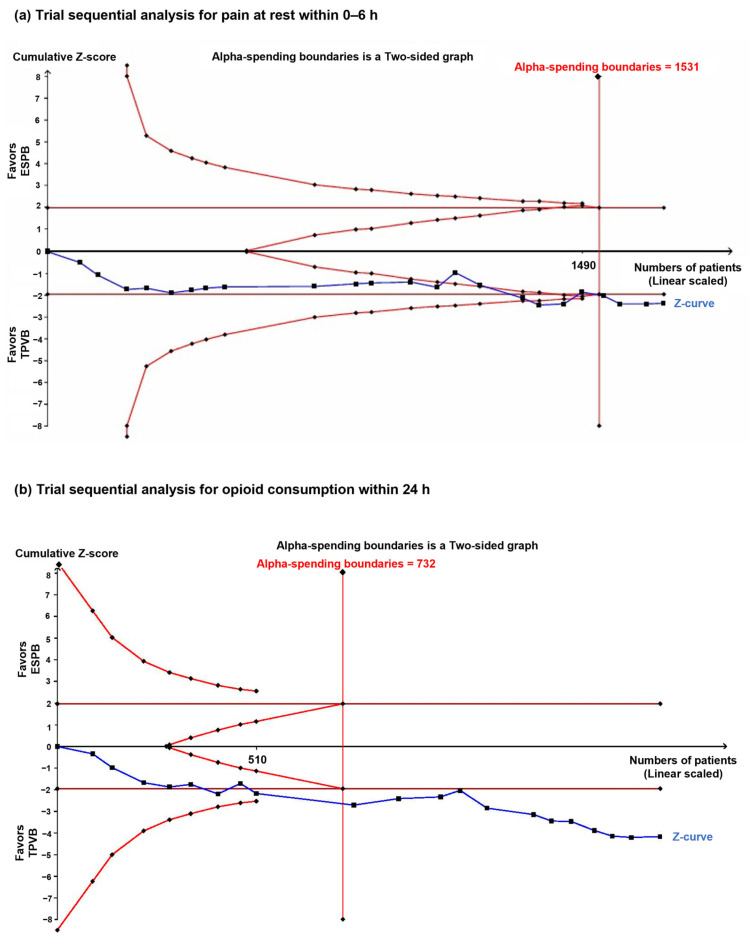
Trial sequential analyses of key postoperative analgesic outcomes. (**a**) Trial sequential analysis for postoperative pain at rest within 0–6 h. (**b**) Trial sequential analysis for cumulative opioid consumption within 24 h. Trial sequential monitoring boundaries were constructed using a two-sided α of 5% and a statistical power of 80%.

TSA, performed only for the RCTs, showed that the cumulative sample size exceeded the RIS (1718 vs. 1537 patients) and that the cumulative Z-curve crossed both the conventional and trial sequential monitoring boundaries (Figure 3a, Table S1).

##### Pain at Rest at 24 h

At postoperative 24 h, 24 studies involving 1768 patients were included, comprising 21 RCTs (1357 patients) [13,14,15,16,17,18,19,20,21,22,24,25,26,27,28,29,30,31,32,33,34] and three OSs (411 patients) [35,36,37]. At 24 h postoperatively, no overall difference in pain at rest was detected between the ESPB and TPVB groups (SMD, 0.077; 95% CI, −0.046 to 0.201; I^2^ = 34.137; P_chi_^2^ = 0.053; τ = 0.170; 95% PI, −0.275 to 0.429). Stratified analyses by study design showed no significant differences in either RCTs (SMD, 0.106; 95% CI, −0.029 to 0.241; I^2^ = 0.0; P_chi_^2^ = 0.641; τ = 0.0) or OSs (SMD, −0.067; 95% CI, −0.371 to 0.237; I^2^ = 88.666; P_chi_^2^ < 0.001; τ = 0.544; 95% PI, −2.408 to 2.274) (Figure S1b, Table S1). Excluding the study by Kukreja et al. [36] led to a loss of statistical significance in the pooled estimate (Figure S2c).

TSA, performed only for the RCTs, showed that the cumulative sample size exceeded the RIS (1468 vs. 650 patients) and that the cumulative Z-curve crossed the futility boundary, suggesting no difference between the ESPB and TPVB groups in postoperative pain at rest at 24 h (Figure S3a, Table S1).

##### Pain at Rest at 48 h

At postoperative 48 h, 12 studies involving 802 patients were included, all of which were RCTs [15,18,19,21,22,24,25,27,28,29,31,33]. Postoperative pain at rest assessed at 48 h did not differ overall between the ESPB and TPVB groups (SMD, −0.154; 95% CI, −0.375 to 0.066; I^2^ = 58.375; P_chi_^2^ = 0.006; τ = 0.294; 95% PI, −0.769 to 0.461) (Figure S1c, Table S1). Excluding the study by Fu et al. [19] resulted in a loss of statistical significance in the pooled estimate.

TSA, performed only for the RCTs, showed that only 21.0% of the RIS was accrued and that the cumulative sample size remained below the RIS (802 vs. 3811 patients); TSA demonstrated that the accumulated evidence failed to meet prespecified thresholds for either conventional significance or trial sequential monitoring, leaving the conclusions inconclusive (Figure S3b, Table S1).

#### 3.3.2. Pain During Cough

##### Overall Pain During Coughing Across All Time Points

A total of 24 studies involving 1789 patients reported pain during coughing, refs. [13,14,15,16,18,19,20,21,22,23,24,25,26,27,28,29,30,31,32,33,34,35,36,37], including 21 RCTs (1378 patients) [13,14,15,16,18,19,20,21,22,23,24,25,26,27,28,29,30,31,32,33,34] and three OSs (411 patients) [35,36,37]. Across postoperative assessments extending from the immediate period through 72 h, no overall difference in pain during coughing was detected between the ESPB and TPVB groups (SMD, 0.140; 95% CI, −0.050 to 0.329; I^2^ = 71.536; P_chi_^2^ < 0.001; τ = 0.383; 95% PI, −0.660 to 0.940). When stratified by study design, no significant differences were observed in either RCTs (SMD, 0.179; 95% CI, −0.025 to 0.383; I^2^ = 71.948; P_chi_^2^ < 0.001; τ = 0.413; 95% PI, −0.681 to 1.039) or OSs (SMD, −0.104; 95% CI, −0.609 to 0.401; I^2^ = 68.683; P_chi_^2^ = 0.041; τ = 0.287; 95% PI, −1.336 to 1.128) (Figure S4a, Table S1). Sensitivity analyses demonstrated that removal of the study by Zengin et al. [30] resulted in a loss of statistical significance (Figure S5a).

##### Early Postoperative Pain During Coughing (0–6 h)

Early postoperative pain during coughing was assessed in 21 studies involving 1603 patients, refs. [13,14,15,16,18,19,21,22,24,25,26,27,28,29,30,31,32,34,35,36,37], including 18 RCTs (1192 patients) [13,14,15,16,18,19,21,22,24,25,26,27,28,29,30,31,32,34] and three OSs (411 patients) [35,36,37]. The pooled analysis demonstrated lower pain scores in the TPVB group compared with the ESPB group; however, substantial heterogeneity was observed (SMD, 0.280; 95% CI, 0.024 to 0.535; I^2^ = 83.755; P_chi_^2^ < 0.001; τ = 0.538; 95% PI, −0.842 to 1.402). When analyses were restricted to RCTs, the difference remained statistically significant (SMD, 0.353; 95% CI, 0.075 to 0.631; I^2^ = 84.555; P_chi_^2^ < 0.001; τ = 0.595; 95% PI, −0.902 to 1.608). In contrast, within OSs, the comparison yielded no significant between-group difference (SMD, −0.127; 95% CI, −0.780 to 0.527; I^2^ = 0.0; P_chi_^2^ = 0.504; τ = 0.0) (Figure S4b, Table S1). Sensitivity analysis showed that exclusion of individual studies led to widening of confidence intervals and occasional loss of statistical significance; however, the direction of the pooled effect consistently favored the TPVB group (Figure S5b) [13,14,15,22,24,32].

TSA, performed only for the RCTs, showed that 52.6% of the RIS was accrued (1192 vs. 2214 patients). The cumulative Z-curve met the criterion for conventional significance but did not satisfy the trial sequential monitoring boundary (Figure S6a, Table S1).

##### Pain During Coughing at 24 h

At postoperative 24 h, 23 studies (1719 patients; 20 RCTs [1308 patients] [13,14,15,16,18,19,20,21,22,24,25,26,27,28,29,31,32,33,34] and three OSs [411 patients] [35,36,37]) were included. No statistically significant between-group difference in cough-related pain was identified in the overall analysis, accompanied by considerable interstudy heterogeneity (SMD, 0.277; 95% CI, −0.052 to 0.606; I^2^ = 90.262; P_chi_^2^ < 0.001; τ = 0.738; 95% PI, −1.250 to 1.804). When stratified by study design, the pooled effect estimate in RCTs was directionally in favor of the TPVB group; however, this finding was accompanied by marked heterogeneity and a wide prediction interval (SMD, 0.389; 95% CI, 0.036 to 0.743; I^2^ = 90.237; P_chi_^2^ < 0.001; τ = 0.787; 95% PI, −1.253 to 2.031). Analyses limited to OSs did not demonstrate a statistically significant difference (SMD, −0.444; 95% CI, −1.338 to 0.450; I^2^ = 90.159; P_chi_^2^ < 0.001; τ = 0.593; 95% PI, −2.995 to 2.107) (Figure S4c, Table S1). Sensitivity analysis did not change statistical significance, with loss of significance after exclusion of the study by Kukreja et al. (Figure S5c) [36].

Consistently, TSA, performed only for the RCTs, showed that 66.4% of the RIS was accrued (1258 vs. 1894 patients), and cumulative Z-curve satisfied the criterion for conventional significance but failed to meet the trial sequential monitoring threshold, leaving the evidence inconclusive (Figure S6b, Table S1).

##### Pain During Coughing at 48 h

At postoperative 48 h, 12 studies including 802 patients (only RCTs) were included [15,18,19,21,22,24,25,27,28,29,31,33]. Pain during coughing assessed at 48 h did not differ between the ESPB and TPVB groups (SMD, −0.017; 95% CI, −0.219 to 0.186; I^2^ = 51.048; P_chi_^2^ = 0.021; τ = 0.253; 95% PI, −0.574 to 0.540) (Figure S4d, Table S1). Leave-one-out sensitivity analyses showed no change in the statistical significance of the pooled estimate, supporting the robustness of the findings (Figure S5d).

TSA, performed only for the RCTs, showed that 7.2% of the RIS was accrued (802 vs. 11,118 patients). The cumulative Z-curve did not satisfy the criteria for either conventional significance or trial sequential monitoring at 48 h, leaving the evidence insufficient for firm conclusions regarding differences between the ESPB and TPVB groups (Figure S6c, Table S1).

#### 3.3.3. Opioid Consumption

##### Overall Opioid Consumption Across Postoperative Period

A total of 24 studies (1764 patients) reported opioid consumption [13,14,15,16,17,18,19,20,21,22,24,25,26,27,28,29,30,31,32,33,34,35,36,37], including 21 RCTs (1353 patients) [13,14,15,16,17,18,19,20,21,22,24,25,26,27,28,29,30,31,32,33,34] and three OSs (411 patients) [35,36,37]. Across all evaluated time points between 6 h and 72 h postoperatively, the pooled results indicated reduced opioid use in the TPVB group relative to the ESPB group (SMD, 0.322; 95% CI, 0.034 to 0.611; I^2^ = 87.541; P_chi_^2^ < 0.001; τ = 0.646; 95% PI, −1.014 to 1.658). Within RCTs, stratified analyses demonstrated reduced opioid consumption in the TPVB group relative to the ESPB group (SMD, 0.358; 95% CI, 0.049 to 0.668; I^2^ = 85.006; P_chi_^2^ < 0.001; τ = 0.614; 95% PI, −0.923 to 1.639). No evidence of a between-group difference was identified in OSs (SMD, 0.083; 95% CI, −0.714 to 0.881; I^2^ = 95.755; P_chi_^2^ < 0.001; τ = 0.940; 95% PI, −3.961 to 4.127) (Figure S7a, Table S1). Sensitivity analysis indicated that the statistical significance of the pooled effect was dependent on the inclusion of individual studies, with loss of significance observed after exclusion of the studies by Chen et al. and Baser et al. (Figure S8a) [14,15].

##### Opioid Consumption at 24 h

Opioid consumption at postoperative 24 h was reported in 20 studies (1545 patients), including 17 RCTs (1143 patients) [13,14,15,16,17,18,19,20,22,24,25,27,28,30,32,33,34] and three OSs (411 patients) [35,36,37]. In the pooled analysis including both RCTs and OSs, opioid consumption at 24 h was lower in the TPVB group than in the ESPB group (SMD, 0.417; 95% CI, 0.108 to 0.725; I^2^ = 87.541; P_chi_^2^ < 0.001; τ = 0.646; 95% PI, −0.935 to 1.769). When restricted to RCTs, the difference remained statistically significant (SMD, 0.438; 95% CI, 0.102 to 0.774; I^2^ = 86.704; P_chi_^2^ < 0.001; τ = 0.650; 95% PI, −0.940 to 1.816), whereas no evidence of a difference was observed in OSs (SMD, 0.303; 95% CI, −0.474 to 1.080; I^2^ = 92.297; P_chi_^2^ < 0.001; τ = 0.672; 95% PI, −2.588 to 3.194) (Figure 2b, Table S1). Sequential exclusion of individual studies did not alter the statistical significance of the pooled effect (Figure S8b).

TSA, performed only for the RCTs, showed that the cumulative sample size exceeded the RIS (1718 vs. 732 patients). The cumulative Z-curve met the criterion for conventional significance but failed to satisfy the trial sequential monitoring boundary, indicating that the findings are not yet definitive (Figure 3b, Table S1).

##### Opioid Consumption at 48 h

At postoperative 48 h, 11 studies (715 patients), all of which were RCTs, were included [15,19,21,24,25,26,27,28,29,31,33]. There was no overall difference in opioid consumption between the ESPB and TPVB groups (SMD, 0.241; 95% CI, −0.112 to 0.595; I^2^ = 81.545; P_chi_^2^ < 0.001; τ = 0.536; 95% PI, −0.953 to 1.435) (Figure S6b, Table S1). Sensitivity analysis demonstrated that the pooled result was robust, with no change in statistical significance after exclusion of individual studies (Figure S7c).

TSA, performed only for the RCTs, showed that only 7.2% of the RIS was accrued (802 vs. 11,118 patients). The cumulative Z-curve failed to reach both the conventional significance threshold and the trial sequential monitoring boundaries, indicating that the evidence was insufficient for definitive conclusions (Figure S9, Table S1).

#### 3.3.4. PONV

A total of 17 studies (1132 patients) reported the incidence of PONV, refs. [13,14,17,18,19,20,22,24,25,26,27,28,30,31,32,35,36] including 15 RCTs (1005 patients) [13,14,17,18,19,20,22,24,25,26,27,28,30,31,32] and two OSs (127 patients) [35,36]. The overall incidence of PONV did not differ between the ESPB and TPVB groups (RR, 0.909; 95% CI, 0.704 to 1.174; I^2^ = 2.372; P_chi_^2^ = 0.426; τ = 0.085; 95% PI, 0.320 to 2.582). Stratified analyses by study design showed no evidence of a difference in PONV incidence between the ESPB and TPVB groups (Figure S10a, Table S1). Sequential exclusion of individual studies in sensitivity analyses did not meaningfully change the pooled effect estimate or its statistical significance (Figure S11a).

TSA, performed only for the RCTs, showed that 47.1% of the required information size (RIS) had been accrued (1005 vs. 2135 patients). As the cumulative Z-curve did not meet the criteria for either conventional significance or trial sequential monitoring, the available evidence remained inadequate to draw definitive conclusions about the effect of ESPB compared with TPVB on PONV (Figure S12a, Table S1).

#### 3.3.5. Hypotension

Ten studies (829 patients) reported the incidence of hypotension, refs. [16,18,21,24,28,30,32,33,34,37] including 9 RCTs (579 patients) [16,18,21,24,28,30,32,33,34] and one OSs (250 patients) [37]. The overall incidence of hypotension did not differ between the ESPB and TPVB groups (RR, 0.680; 95% CI, 0.359 to 1.291; I^2^ = 41.119; P_chi_^2^ = 0.083; τ = 0.510; 95% PI, 0.198 to 2.337). In subgroup analyses according to study design, no significant difference was observed in RCTs (RR, 0.574; 95% CI, 0.279 to 1.184; I^2^ = 40.314; P_chi_^2^ = 0.099; τ = 0.636; 95% PI, 0.247 to 1.331). The single OS did not demonstrate evidence of a between-group difference (RR, 1.257; 95% CI, 0.703 to 2.249) (Figure S10b, Table S1). The direction and statistical significance of the pooled effect estimate remained unchanged across leave-one-out sensitivity analyses (Figure S11b).

TSA, performed only for the RCTs, indicated that only 13.4% of the RIS had been accrued (579 vs. 4317 patients). As the cumulative Z-curve failed to satisfy both the conventional boundary and the trial sequential monitoring criteria, the evidence remains inadequate to establish a definitive effect on hypotension (Figure S12b, Table S1).

### 3.4. Publication Bias Assessment

For 13 outcomes comprising 10 or more studies, funnel plots were examined and demonstrated an approximately symmetric distribution of studies around the pooled estimates across all assessed outcomes (Figure S13a–m, Supplementary Materials, Document S1).

Formal statistical assessment did not identify evidence of publication bias for any outcome when regression-based and rank correlation–based methods were applied, including pain at rest (overall, early postoperative, 24 h, and 48 h), pain during coughing (overall, early postoperative, 24 h, and 48 h), cumulative opioid consumption (overall, 24 h, and 48 h), PONV, and hypotension (all *p* > 0.05).

Detailed results of the publication bias analyses, including bias coefficients, confidence intervals, and test statistics, are provided in the Figure S13a–m, Supplementary Materials, Document S1.

### 3.5. Certainty of Evidence

Using the GRADE approach, 28 outcomes were assessed across the 25 included studies (Table S2). The certainty of evidence was high for postoperative pain at rest at 24 h, PONV, and hypotension, whereas most other pain- and opioid-related outcomes were of moderate certainty. Evidence derived from OSs was generally rated as low to very low certainty.

## 4. Discussion

Drawing on evidence from 25 studies including 1847 patients, this systematic review and meta-analysis examines postoperative analgesic outcomes associated with ESPB and TPVB following thoracic surgery. The principal finding is that TPVB provides superior analgesia during the early postoperative period, whereas analgesic efficacy converges thereafter, with no clinically meaningful differences between the two techniques at 24 or 48 h postoperatively. In pooled analyses including both RCTs and OSs, opioid consumption at 24 h was modestly lower with TPVB; however, this finding was characterized by substantial heterogeneity and low certainty of evidence. Importantly, ESPB and TPVB demonstrated comparable safety profiles, with no significant differences in postoperative nausea and vomiting or hypotension.

The early postoperative analgesic advantage observed with TPVB is biologically plausible and consistent with the anatomical characteristics of the block [38,39]. TPVB involves direct deposition of local anesthetic into the paravertebral space, allowing immediate and predictable spread to the spinal nerves, dorsal root ganglia, and sympathetic chain [40]. This results in dense segmental somatic and sympathetic blockade, which is particularly relevant in the immediate postoperative period when nociceptive input is greatest [3]. In contrast, ESPB relies on indirect diffusion of local anesthetic from the erector spinae muscle plane into the paravertebral space through fascial planes and foraminal pathways [7,8]. This indirect mechanism may lead to more variable spread and slower onset of analgesia, likely accounting for the observed differences in early pain scores [41]. The robustness of this early benefit is supported by trial sequential analysis, which demonstrated that cumulative evidence for early pain at rest crossed both the conventional monitoring boundary and the required information size, indicating that the superiority of TPVB during this narrow time window is unlikely to be attributable to random error [42]. However, the absolute magnitude of the observed early pain difference appears limited. An effect size of approximately 0.25 corresponds to an absolute reduction of roughly 0.5–0.8 points on a 10-point VAS or NRS scale, assuming commonly reported postoperative pain variability [43,44]. This magnitude is close to or below the minimal clinically important difference of approximately 1 point for acute postoperative pain, indicating that the statistically significant early advantage is modest in clinical terms for most patients [44,45,46].

Although TPVB showed a consistent advantage in early postoperative analgesia, substantial heterogeneity was observed for pain outcomes within 0–6 h and for opioid consumption at 24 h. This heterogeneity was largely confined to the early postoperative period, when analgesic effects are more strongly influenced by block-specific characteristics than by standardized multimodal analgesic regimens applied later [47]. ESPB relies on indirect diffusion of local anesthetic from the erector spinae muscle plane, which may result in variable early analgesic effects depending on individual tissue characteristics and diffusion patterns, whereas TPVB involves direct injection into the paravertebral space and is more likely to produce consistent segmental blockade [41,48,49]. In addition, opioid-related outcomes are inherently sensitive to differences in postoperative analgesic protocols, rescue thresholds, and opioid conversion methods across studies, which may further contribute to between-study heterogeneity [50]. Recent experimental data further suggest that local anesthetics such as bupivacaine may spread anisotropically within muscle and traverse fascial boundaries via diffusion-dominated mechanisms that are not readily apparent on imaging [51]. Such imaging-invisible diffusion may contribute to variable and occasionally discordant early analgesic effects in transfascial plane blocks such as ESPB and may partly explain the observed heterogeneity across studies. Accordingly, interpretation of these outcomes should be guided primarily by evidence from RCTs, with findings from OSs considered supportive given their higher risk of bias.

Beyond the early postoperative period, analgesic efficacy converged between ESPB and TPVB. At 24 and 48 h postoperatively, no significant differences were observed in pain scores at rest or during coughing. This convergence likely reflects several factors. First, the pharmacological effects of single-injection regional blocks diminish over time, reducing differences attributable to block-specific characteristics [52]. Second, postoperative pain control beyond the immediate recovery phase is predominantly influenced by multimodal analgesia, including systemic non-opioid and opioid medications, which may attenuate differences between regional techniques [53,54]. Prediction intervals for pain outcomes at later time points crossed the line of no effect, indicating that future studies could plausibly favor either technique depending on patient characteristics, surgical factors, and institutional protocols [55]. Sensitivity analyses confirmed that these findings were stable and not driven by individual studies, supporting the interpretation that ESPB and TPVB provide comparable analgesia beyond the immediate postoperative phase when integrated into contemporary multimodal analgesic pathways [56].

With respect to opioid consumption, TPVB was associated with lower opioid use at 24 h postoperatively; however, this finding warrants cautious interpretation. This pooled estimate was influenced by OSs, all of which were assessed as having a high risk of bias, and trial sequential analysis indicated insufficient information size to support firm conclusions. The certainty of evidence was rated as low due to substantial heterogeneity across studies, likely reflecting differences in opioid conversion methods, patient-controlled analgesia protocols, rescue analgesic thresholds, and baseline patient characteristics [50]. Trial sequential analysis further showed that the cumulative evidence for opioid consumption did not reach the required information size, indicating that a sustained opioid-sparing advantage of TPVB could not be confirmed [42]. Moreover, the magnitude of the observed difference was modest and may not translate into clinically meaningful reductions in opioid-related adverse effects within contemporary multimodal analgesia frameworks [47]. These considerations underscore the importance of distinguishing statistical significance from clinical relevance when interpreting opioid-related outcomes [57].

Both ESPB and TPVB demonstrated favorable and comparable safety profiles. The incidence of postoperative nausea and vomiting and hypotension was comparable between the ESPB and TPVB groups, with the certainty of evidence for these outcomes ranging from moderate to high. These findings are reassuring, particularly given historical concerns regarding sympathetic blockade and hemodynamic instability associated with TPVB [6]. Nevertheless, the available evidence remains underpowered to assess rare but potentially serious complications such as pneumothorax, local anesthetic systemic toxicity, or nerve injury [49]. Although ESPB is often perceived as safer due to its more superficial target and greater distance from critical structures, definitive comparative safety conclusions cannot be drawn based on the current evidence base [58].

The present findings are consistent with and extend those of previous systematic reviews and meta-analyses comparing ESPB and TPVB for thoracic surgery. Recent meta-analyses published between 2023 and 2024 similarly reported no clinically meaningful differences in pain outcomes beyond the early postoperative period, while noting early analgesic advantages for TPVB in some analyses [9,10,59,60]. The consistency of findings across multiple independent reviews strengthens confidence that the observed patterns reflect genuine clinical effects rather than methodological artifacts. Importantly, this study contributes novel insights by providing the most comprehensive application of the GRADE approach to date, demonstrating moderate-certainty evidence for pain outcomes and low-certainty evidence for opioid outcomes due to inconsistency and imprecision [61]. In addition, this analysis represents the first systematic evaluation of publication bias across all eligible outcomes using both visual inspection and statistical testing, with no evidence of selective reporting identified [62]. The incorporation of trial sequential analysis further clarifies which conclusions are supported by sufficient evidence and which remain uncertain [42].

From a clinical perspective, these findings support a nuanced approach to regional analgesia selection for thoracic surgery. TPVB may be preferred when superior early postoperative analgesia is prioritized, particularly in patients for whom early pain control is critical to facilitate respiratory function and mobilization [63]. Conversely, ESPB represents a reasonable alternative when technical simplicity, ease of performance, or resource considerations are emphasized [17,32]. Emerging evidence suggests that ESPB may offer practical advantages, including shorter block performance time and potentially fewer technical failures, particularly in real-world settings or among less experienced practitioners [13,64]. These considerations are increasingly relevant in high-volume surgical centers and institutions implementing regional anesthesia programs [65]. Nonetheless, the choice between ESPB and TPVB should be individualized and informed not only by analgesic efficacy but also by operator experience, patient anatomy, surgical approach, and institutional protocols [66].

Several limitations of this meta-analysis merit consideration. The risk of bias was moderate in many included randomized trials, primarily due to lack of blinding, which may have influenced subjective pain assessments [67,68]. Substantial heterogeneity was observed for several outcomes, particularly opioid consumption, limiting the precision of pooled estimates [56]. The inclusion of observational studies, while enhancing external validity, introduces potential confounding by indication and limits their contribution to supportive rather than confirmatory evidence [69]. Trial sequential analysis indicated that the evidence base remains insufficient for several outcomes, including opioid consumption and later pain assessments, precluding definitive conclusions regarding equivalence or sustained superiority [42]. Furthermore, most included studies focused on short-term outcomes, with limited reporting of long-term pain, functional recovery, or quality of life [1,2]. Although publication bias was not detected, small-study effects cannot be entirely excluded [62].

## 5. Conclusions

Overall, the findings indicate that ESPB and TPVB achieve comparable postoperative analgesic effects after thoracic surgery beyond the immediate postoperative period, with moderate certainty of evidence. TPVB demonstrates superior analgesia within the first 6 h postoperatively, supported by firm evidence from trial sequential analysis, but this advantage does not persist at later time points. In pooled analyses including both RCTs and OSs, evidence for opioid-sparing effects of TPVB at 24 h remains uncertain due to heterogeneity and insufficient information size. Both techniques exhibit comparable safety profiles. ESPB may therefore be considered a practical and effective alternative to TPVB within multimodal analgesia strategies, while TPVB may be favored when early postoperative analgesia is prioritized. Individualized selection based on patient characteristics, operator expertise, and institutional context remains essential, and further high-quality studies are required to address remaining evidence gaps.

## Figures and Tables

**Table 1:** Overview of studies included in the meta-analysis.

Study	Country	Study Design	ESPB Group (*n*)	TPVB Group (*n*)	Surgery Type
Andrade Filho et al. 2024 [13]	Brazil	RCT	61	59	Mixed
Baser et al. 2025 [14]	Turkey	RCT	30	30	Thoracotomy
Chen et al. 2020 [15]	China	RCT	25	25	Thoracoscopy
Das et al. 2022 [16]	India	RCT	30	28	Thoracotomy
Duran et al. 2024 [17]	Turkey	RCT	23	22	Thoracotomy
Durey et al. 2023 [35]	France	OS	54	53	Thoracoscopy
Fang et al. 2019 [18]	China	RCT	45	46	Thoracotomy
Fu et al. 2022 [19]	China	RCT	20	20	Thoracoscopy
Geyik et al. 2024 [20]	Turkey	RCT	25	25	Thoracotomy
Kim et al. 2022 [21]	S. Korea	RCT	26	26	Thoracoscopy
Kukreja et al. 2021 [36]	USA	OS	20	34	Mixed
Li et al. 2025 [22]	China	RCT	22	24	Thoracoscopy
Mogahed et al. 2024 [23]	Egypt	RCT	35	35	Thoracoscopy
Moorthy et al. 2023 [24]	Ireland	RCT	37	37	Thoracoscopy
Sertcakacilar et al. 2022 [37]	Turkey	OS	136	114	Thoracoscopy
Sun et al. 2022 [25]	China	RCT	59	56	Thoracoscopy
Sun et al. 2024 [26]	China	RCT	25	25	Thoracoscopy
Taketa et al. 2019 [27]	Japan	RCT	41	40	Thoracoscopy
Turhan et al. 2021 [28]	Turkey	RCT	35	35	Thoracoscopy
Wang et al. 2025 [29]	China	RCT	36	37	Thoracoscopy
Zengin et al. 2023 [30]	Turkey	RCT	25	25	Thoracoscopy
Zhang et al. 2022 [31]	China	RCT	22	22	Thoracoscopy
Zhang et al. 2023 [32]	China	RCT	33	36	Thoracoscopy
Zhao et al. 2020 [33]	China	RCT	33	33	Thoracoscopy
Zhu et al. 2025 [34]	China	RCT	24	25	Thoracoscopy

‘Mixed’ indicates that the study included patients undergoing different types of thoracic surgery without stratification by surgical approach. ESPB, erector spinae plane block; OS, observational study; RCT, randomized controlled trial; S. Korea, South Korea; TPVB, thoracic paravertebral block.

**Table 2:** Risk of bias assessment of included RCTs (Cochrane RoB 2).

Study	Randomization Process	Deviations from Intended Interventions	Missing Outcome Data	Measurement of the Outcome	Selection of the Reported Result	Overall Bias
Andrade Filho et al. 2024 [13]	Low risk	Some concerns ^b^	Low risk	Some concerns ^c^	Low risk	Some concerns
Baser et al. 2025 [14]	Some concerns ^a^	Some concerns ^b^	Low risk	Some concerns ^c^	Some concerns ^d^	Some concerns
Chen et al. 2020 [15]	Low risk	Some concerns ^b^	Low risk	Some concerns ^c^	Some concerns ^d^	Some concerns
Das et al. 2022 [16]	Low risk	Some concerns ^b^	Low risk	Some concerns ^c^	Some concerns ^d^	Some concerns
Duran et al. 2024 [17]	Low risk	Some concerns ^b^	Low risk	Some concerns ^c^	Low risk	Some concerns
Durey et al. 2023 [35]	Low risk	Some concerns ^b^	Low risk	Some concerns ^c^	Low risk	Some concerns
Fang et al. 2019 [18]	Low risk	Low risk	Low risk	Low risk	Low risk	Low risk
Fu et al. 2022 [19]	Some concerns ^a^	Some concerns ^b^	Low risk	Some concerns ^c^	Some concerns ^d^	Some concerns
Geyik et al. 2024 [20]	Low risk	Some concerns ^b^	Low risk	Low risk	Low risk	Some concerns
Kim et al. 2022 [21]	Low risk	Low risk	Low risk	Low risk	Low risk	Low risk
Kukreja et al. 2021 [36]	Some concerns ^a^	Some concerns ^b^	Low risk	Some concerns ^c^	Some concerns ^d^	Some concerns
Li et al. 2025 [22]	Low risk	Low risk	Low risk	Low risk	Low risk	Low risk
Mogahed et al. 2024 [23]	Low risk	Some concerns ^b^	Low risk	Some concerns ^c^	Low risk	Some concerns
Moorthy et al. 2023 [24]	Low risk	Some concerns ^b^	Low risk	Some concerns ^c^	Low risk	Some concerns
Sertcakacilar et al. 2022 [37]	Low risk	Some concerns ^b^	Low risk	Some concerns ^c^	Low risk	Some concerns
Sun et al. 2022 [25]	Low risk	Some concerns ^b^	Low risk	Low risk	Low risk	Some concerns
Sun et al. 2024 [26]	Low risk	Some concerns ^b^	Low risk	Low risk	Low risk	Some concerns
Taketa et al. 2019 [27]	Low risk	Some concerns ^b^	Low risk	Low risk	Low risk	Some concerns
Turhan et al. 2021 [28]	Low risk	Some concerns ^b^	Low risk	Some concerns ^c^	Low risk	Some concerns
Wang et al. 2025 [29]	Low risk	Some concerns ^b^	Low risk	Some concerns ^c^	Low risk	Some concerns
Zengin et al. 2023 [30]	Low risk	Some concerns ^b^	Low risk	Some concerns ^c^	Low risk	Some concerns
Zhang et al. 2022 [31]	Low risk	Low risk	Low risk	Low risk	Low risk	Low risk
Zhang et al. 2023 [32]	Low risk	Some concerns ^b^	Low risk	Some concerns ^c^	Low risk	Some concerns
Zhao et al. 2020 [33]	Some concerns ^a^	Some concerns ^b^	Low risk	Some concerns ^c^	Some concerns ^d^	Some concerns
Zhu et al. 2025 [34]	Low risk	Some concerns ^b^	Low risk	Some concerns ^c^	Some concerns ^d^	Some concerns

^a^: no information was provided on how allocation concealment was performed, ^b^: participants and/or study personnel were aware of the assigned intervention, and no information was reported on whether any deviations occurred as a result of the trial context, ^c^: no information was provided on whether outcome assessors were blinded to the intervention received, and knowledge of the intervention may have influenced the assessment of outcomes, ^d^: no details were provided regarding adherence to a prespecified analysis plan.

**Table 3:** Risk of bias assessment of included non-randomized studies (RoBANS).

Study	Selection of Participants	Confounding Variables	Intervention Measurement	Blinding of Outcome Assessment	Incomplete Outcome Data	Selective Outcome Reporting	Overall RoB
Durey et al. 2023 [35]	Low risk	Low risk	Low risk	High risk ^c^	Low risk	Unclear risk ^d^	High risk
Kukreja et al. 2021 [36]	Unclear risk ^a^	High risk ^b^	Low risk	High risk ^c^	Low risk	Unclear risk ^d^	High risk
Sertcakacilar et al. 2022 [37]	Unclear risk ^a^	High risk ^b^	Low risk	High risk ^c^	Low risk	Unclear risk ^d^	High risk

^a^: allocation based on clinician preference or institutional protocols, ^b^: inappropriate confounder confirmation and consideration, ^c^: outcome assessors were not blinded, ^d^: study protocol was not registered.

## Data Availability

The data supporting the findings of this study were obtained from publicly available published articles and their Supplementary Materials. Extracted data used in the meta-analysis are available from the corresponding author upon reasonable request.

## Supplementary Materials

The following supporting information can be downloaded at: https://www.mdpi.com/article/10.3390/jcm15041370/s1, File S1: PRISMA 2020 Checklist; File S2: Detailed search strategy and search terms; Figure S1: Postoperative pain at rest; Figure S2: Sensitivity analysis for pain at rest; Figure S3: Postoperative pain during coughing; Figure S4: Sensitivity analysis for pain during coughing; Figure S5: Postoperative opioid consumption; Figure S6: Sensitivity analysis for opioid consumption; Figure S7: Postoperative nausea and vomiting; Figure S8: Postoperative hypotension; Figure S9: Publication bias for pain at rest; Figure S10: Publication bias for pain during coughing; Figure S11: Publication bias for opioid consumption; Figure S12: Trial sequential analyses; Table S1: Summary of Meta-analysis and Trial Sequential Analysis; Table S2: Certainty of Evidence Assessment Using GRADE; Document S1: Publication Bias Assessment Including Figure S13.

## Abbreviations

The following abbreviations are used in this manuscript:

CENTRAL	Cochrane Central Register of Controlled Trials
CI	Confidence Interval
ERAS	Enhanced Recovery After Surgery
ESP	Erector Spinae Plane
ESPB	Erector Spinae Plane Block
ESTS	European Society of Thoracic Surgeons
GRADE	Grading of Recommendations Assessment, Development and Evaluation
NRS	Numerical Rating Scale
OME	Oral Morphine Equivalents
OR	Odds Ratio
OS	Observational Study
PI	Prediction Interval
PICOS	Population, Intervention, Comparison, Outcome, Study Design
PONV	Postoperative Nausea and Vomiting
PRISMA	Preferred Reporting Items for Systematic Reviews and Meta-Analyses
PVB	Paravertebral Block
RCT	Randomized Controlled Trial
RIS	Required Information Size
RR	Risk Ratio
RoB	Risk of Bias
SMD	Standardized Mean Difference
TPVB	Thoracic Paravertebral Block
TSA	Trial Sequential Analysis
VAS	Visual Analog Scale
VATS	Video-Assisted Thoracoscopic Surgery

## References

[1] Kehlet H., Jensen T.S., Woolf C.J. (2006). Persistent postsurgical pain: Risk factors and prevention. Lancet.

[2] Wildgaard K., Ravn J., Kehlet H. (2009). Chronic post-thoracotomy pain: A critical review of pathogenic mechanisms and strategies for prevention. Eur. J. Cardio-Thorac. Surg..

[3] Joshi G.P., Bonnet F., Shah R., Wilkinson R.C., Camu F., Fischer B., Neugebauer E.A., Rawal N., Schug S.A., Simanski C. (2008). A systematic review of randomized trials evaluating regional techniques for postthoracotomy analgesia. Anesth. Analg..

[4] Karmakar M.K. (2001). Thoracic paravertebral block. Anesthesiology.

[5] Davies R.G., Myles P.S., Graham J.M. (2006). A comparison of the analgesic efficacy and side-effects of paravertebral vs epidural blockade for thoracotomy—A systematic review and meta-analysis of randomized trials. Br. J. Anaesth..

[6] Pace M.M., Sharma B., Anderson-Dam J., Fleischmann K., Warren L., Stefanovich P. (2016). Ultrasound-Guided Thoracic Paravertebral Blockade: A Retrospective Study of the Incidence of Complications. Anesth. Analg..

[7] Forero M., Adhikary S.D., Lopez H., Tsui C., Chin K.J. (2016). The Erector Spinae Plane Block: A Novel Analgesic Technique in Thoracic Neuropathic Pain. Reg. Anesth. Pain Med..

[8] Kot P., Rodriguez P., Granell M., Cano B., Rovira L., Morales J., Broseta A., Andres J. (2019). The erector spinae plane block: A narrative review. Korean J. Anesthesiol..

[9] Capuano P., Hileman B.A., Martucci G., Raffa G.M., Toscano A., Burgio G., Arcadipane A., Kowalewski M. (2023). Erector spinae plane block versus paravertebral block for postoperative pain management in thoracic surgery: A systematic review and meta-analysis. Minerva Anestesiol..

[10] Fenta E., Kibret S., Hunie M., Tamire T., Mekete G., Tiruneh A., Fentie Y., Dessalegn K., Teshome D. (2023). The analgesic efficacy of erector spinae plane block versus paravertebral block in thoracic surgeries: A meta-analysis. Front. Med..

[11] Higgins J.P., Sterne J.A., Savović J., Page M.J., Hrobjartsson A., Boutron I., Reeves B., Eldridge S. (2016). A revised tool for assessing risk of bias in randomized trials. Cochrane Database Syst. Rev..

[12] Kim S.Y., Park J.E., Lee Y.J., Seo H.J., Sheen S.S., Hahn S., Jang B.H., Son H.J. (2013). Testing a tool for assessing the risk of bias for nonrandomized studies showed moderate reliability and promising validity. J. Clin. Epidemiol..

[13] Andrade Filho P.H., Pereira V.E., Sousa D., Costa L.D.G., Nunes Y.P., Taglialegna G., de Paula-Garcia W.N., Silva J.M. (2024). Analgesic efficacy of erector spinae plane block versus paravertebral block in lung surgeries-A non-inferiority randomised controlled trial. Acta Anaesthesiol. Scand..

[14] Baser K., Adiyeke O., Mendes E., Gumus Ozcan F. (2025). Optimizing post-thoracotomy pain management: Comparing erector spinae vs. paravertebral block in thoracotomy patients: A prospective randomized study. BMC Anesth..

[15] Chen N., Qiao Q., Chen R., Xu Q., Zhang Y., Tian Y. (2020). The effect of ultrasound-guided intercostal nerve block, single-injection erector spinae plane block and multiple-injection paravertebral block on postoperative analgesia in thoracoscopic surgery: A randomized, double-blinded, clinical trial. J. Clin. Anesth..

[16] Das S., Saha D., Sen C. (2022). Comparison Among Ultrasound-Guided Thoracic Paravertebral Block, Erector Spinae Plane Block and Serratus Anterior Plane Block for Analgesia in Thoracotomy for Lung Surgery. J. Cardiothorac. Vasc. Anesth..

[17] Duran M., Kus A., Aksu C., Cesur S., Yorukoglu H.U., Hosten T. (2024). Comparison of Postoperative Opioid Consumption of Paravertebral Block and Erector Spinae Plane Block After Thoracotomy: A Randomized Controlled Trial. Cureus.

[18] Fang B., Wang Z., Huang X. (2019). Ultrasound-guided preoperative single-dose erector spinae plane block provides comparable analgesia to thoracic paravertebral block following thoracotomy: A single center randomized controlled double-blind study. Ann. Transl. Med..

[19] Fu Z., Zhang Y., Zhou Y., Li Z., Wang K., Li H., Jiang W., Liu Z., Cao X. (2022). A comparison of paravertebral block, erector spinae plane block and the combination of erector spinae plane block and paravertebral block for post-operative analgesia after video-assisted thoracoscopic surgery: A randomised controlled trial. J. Minim. Access Surg..

[20] Geyik F.D., Arslan G., Sayın Kart J., Dalkılınç Hökenek U., Doğruyol M.T., Demirhan R., Saracoğlu K.T. (2024). Comparison of Four Different Block Techniques for Postoperative Analgesia in Thoracotomy. GKDA Derg..

[21] Kim S., Song S.W., Do H., Hong J., Byun C.S., Park J.H. (2022). The Analgesic Efficacy of the Single Erector Spinae Plane Block with Intercostal Nerve Block Is Not Inferior to That of the Thoracic Paravertebral Block with Intercostal Nerve Block in Video-Assisted Thoracic Surgery. J. Clin. Med..

[22] Li J., Shao P., Wen H., Ma D., Yang L., He J., Jiang J. (2025). Comparison of Costotransverse Foramen Block with Thoracic Paravertebral Block and Erector Spinae Plane Block for Patients Undergoing Video-Assisted Thoracoscopic Surgery: A Randomized Controlled Non-Inferiority Trial. J. Pain Res..

[23] Mohamed Mogahed M., Abd El-ghaffar M.S., Al Noamani T.S., Elkahwagy M.S. (2024). Erector spinae plane versus paravertebral versus multiple intercostal nerve blocks in patients undergoing vats; A randomized controlled trial. Perioper. Care Oper. Room Manag..

[24] Moorthy A., Ni Eochagain A., Dempsey E., Wall V., Marsh H., Murphy T., Fitzmaurice G.J., Naughton R.A., Buggy D.J. (2023). Postoperative recovery with continuous erector spinae plane block or video-assisted paravertebral block after minimally invasive thoracic surgery: A prospective, randomised controlled trial. Br. J. Anaesth..

[25] Sun L., Mu J., Gao B., Pan Y., Yu L., Liu Y., He H. (2022). Comparison of the efficacy of ultrasound-guided erector spinae plane block and thoracic paravertebral block combined with intercostal nerve block for pain management in video-assisted thoracoscopic surgery: A prospective, randomized, controlled clinical trial. BMC Anesth..

[26] Sun L., Mu J., Yu L., Hu J., Hu Y., He H. (2024). Continuous Erector Spinae Plane Block for Postoperative Analgesia in Elderly Patients After Thoracoscopic Lobectomy. J. Perianesth Nurs..

[27] Taketa Y., Irisawa Y., Fujitani T. (2019). Comparison of ultrasound-guided erector spinae plane block and thoracic paravertebral block for postoperative analgesia after video-assisted thoracic surgery: A randomized controlled non-inferiority clinical trial. Reg. Anesth. Pain Med..

[28] Turhan O., Sivrikoz N., Sungur Z., Duman S., Ozkan B., Senturk M. (2021). Thoracic Paravertebral Block Achieves Better Pain Control Than Erector Spinae Plane Block and Intercostal Nerve Block in Thoracoscopic Surgery: A Randomized Study. J. Cardiothorac. Vasc. Anesth..

[29] Wang L.F., Qi F., Feng H.X., Shi Y.H., Li Y., Zheng M.T., Bu T., Li W.X., Zhang Z.R. (2025). Risk and benefit analysis of single-shot nerve block for postoperative analgesia for uniportal video-assisted thoracic surgery (uVATS): A randomized controlled trial. BMC Anesth..

[30] Zengin M., Alagoz A., Sazak H., Ulger G., Baldemir R., Senturk M. (2023). Comparison of efficacy of erector spinae plane block, thoracic paravertebral block, and erector spinae plane block and thoracic paravertebral block combination for acute pain after video-assisted thoracoscopic surgery: A randomized controlled study. Minerva Anestesiol..

[31] Zhang J.W., Feng X.Y., Yang J., Wang Z.H., Wang Z., Bai L.P. (2022). Ultrasound-guided single thoracic paravertebral nerve block and erector spinae plane block for perioperative analgesia in thoracoscopic pulmonary lobectomy: A randomized controlled trial. Insights Imaging.

[32] Zhang L., Hu Y., Liu H., Qi X., Chen H., Cao W., Wang L., Zhang Y., Wu Y. (2023). Analgesic Efficacy of Combined Thoracic Paravertebral Block and Erector Spinae Plane Block for Video-Assisted Thoracic Surgery: A Prospective Randomized Clinical Trial. Med. Sci. Monit..

[33] Zhao H., Xin L., Feng Y. (2020). The effect of preoperative erector spinae plane vs. paravertebral blocks on patient-controlled oxycodone consumption after video-assisted thoracic surgery: A prospective randomized, blinded, non-inferiority study. J. Clin. Anesth..

[34] Zhu Y., Yang Y., Zhang Q., Li X., Xue W., Liu Y., Zhao Y., Xu W., Yan P., Li S. (2025). Comparison of Ultrasound-guided Single-injection Erector Spinae Plane Block, Retrolaminar Block, and Paravertebral Block for Postoperative Analgesia in Single-incision Video-assisted Thoracoscopic Surgery: A 3-arm, Double-blind, Randomized Controlled Noninferiority Trial. Clin. J. Pain.

[35] Durey B., Djerada Z., Boujibar F., Besnier E., Montagne F., Baste J.M., Dusseaux M.M., Compere V., Clavier T., Selim J. (2023). Erector Spinae Plane Block versus Paravertebral Block after Thoracic Surgery for Lung Cancer: A Propensity Score Study. Cancers.

[36] Kukreja P., Herberg T.J., Johnson B.M., Kofskey A.M., Short R.T., MacBeth L., Paul C., Kalagara H. (2021). Retrospective Case Series Comparing the Efficacy of Thoracic Epidural With Continuous Paravertebral and Erector Spinae Plane Blocks for Postoperative Analgesia After Thoracic Surgery. Cureus.

[37] Sertcakacilar G., Pektas Y., Yildiz G.O., Isgorucu O., Kose S. (2022). Efficacy of ultrasound-guided erector spinae plane block versus paravertebral block for postoperative analgesia in single-port video-assisted thoracoscopic surgery: A retrospective study. Ann. Palliat. Med..

[38] Karmakar M.K., Samy W., Li J.W., Lee A., Chan W.C., Chen P.P., Ho A.M. (2014). Thoracic paravertebral block and its effects on chronic pain and health-related quality of life after modified radical mastectomy. Reg. Anesth. Pain Med..

[39] Tighe S.Q.M., Greene M.D., Rajadurai N. (2010). Paravertebral block. Contin. Educ. Anaesth. Crit. Care Pain.

[40] Richardson J., Lönnqvist P.A., Naja Z. (2011). Bilateral thoracic paravertebral block: Potential and practice. Br. J. Anaesth..

[41] Schwartzmann A., Peng P., Maciel M.A., Forero M. (2018). Mechanism of the erector spinae plane block: Insights from a magnetic resonance imaging study. Can. J. Anaesth..

[42] Wetterslev J., Thorlund K., Brok J., Gluud C. (2008). Trial sequential analysis may establish when firm evidence is reached in cumulative meta-analysis. J. Clin. Epidemiol..

[43] Olsen M.F., Bjerre E., Hansen M.D., Hilden J., Landler N.E., Tendal B., Hrobjartsson A. (2017). Pain relief that matters to patients: Systematic review of empirical studies assessing the minimum clinically important difference in acute pain. BMC Med..

[44] Myles P.S., Myles D.B., Galagher W., Chew C., MacDonald N., Dennis A. (2016). Minimal Clinically Important Difference for Three Quality of Recovery Scales. Anesthesiology.

[45] Cepeda M.S., Africano J.M., Polo R., Alcala R., Carr D.B. (2003). What decline in pain intensity is meaningful to patients with acute pain?. Pain.

[46] Gewandter J.S., Eisenach J.C., Gross R.A., Jensen M.P., Keefe F.J., Lee D.A., Turk D.C. (2019). Checklist for the preparation and review of pain clinical trial publications: A pain-specific supplement to CONSORT. Pain Rep..

[47] Joshi G.P., Kehlet H. (2013). Procedure-specific pain management: The road to improve postsurgical pain management?. Anesthesiology.

[48] Tulgar S., Selvi O., Kapakli M.S. (2018). Erector Spinae Plane Block for Different Laparoscopic Abdominal Surgeries: Case Series. Case Rep. Anesth..

[49] Krediet A.C., Moayeri N., van Geffen G.J., Bruhn J., Renes S., Bigeleisen P.E., Groen G.J. (2015). Different Approaches to Ultrasound-guided Thoracic Paravertebral Block: An Illustrated Review. Anesthesiology.

[50] Nielsen S., Degenhardt L., Hoban B., Gisev N. (2016). A synthesis of oral morphine equivalents (OME) for opioid utilisation studies. Pharmacoepidemiol. Drug Saf..

[51] Istenič S., Pušnik L., Kenneth Ugwoke C., Stopar Pintarič T., Umek N. (2026). Mechanistic insights into bupivacaine spread through anisotropic tissue planes and fascial barriers: Experimental evidence for interfascial block dynamics. Reg. Anesth. Pain Med..

[52] Hutchins J., Apostolidou I., Shumway S., Kelly R., Wang Q., Foster C., Loor G. (2017). Paravertebral Catheter Use for Postoperative Pain Control in Patients After Lung Transplant Surgery: A Prospective Observational Study. J. Cardiothorac. Vasc. Anesth..

[53] Wick E.C., Grant M.C., Wu C.L. (2017). Postoperative Multimodal Analgesia Pain Management With Nonopioid Analgesics and Techniques: A Review. JAMA Surg..

[54] Chou R., Gordon D.B., de Leon-Casasola O.A., Rosenberg J.M., Bickler S., Brennan T., Carter T., Cassidy C.L., Chittenden E.H., Degenhardt E. (2016). Management of Postoperative Pain: A Clinical Practice Guideline From the American Pain Society, the American Society of Regional Anesthesia and Pain Medicine, and the American Society of Anesthesiologists’ Committee on Regional Anesthesia, Executive Committee, and Administrative Council. J. Pain.

[55] IntHout J., Ioannidis J.P., Rovers M.M., Goeman J.J. (2016). Plea for routinely presenting prediction intervals in meta-analysis. BMJ Open.

[56] Higgins J.P., Thompson S.G., Deeks J.J., Altman D.G. (2003). Measuring inconsistency in meta-analyses. BMJ.

[57] Guyatt G.H., Oxman A.D., Kunz R., Woodcock J., Brozek J., Helfand M., Alonso-Coello P., Glasziou P., Jaeschke R., Akl E.A. (2011). GRADE guidelines: 7. Rating the quality of evidence--inconsistency. J. Clin. Epidemiol..

[58] El-Boghdadly K., Pawa A. (2017). The erector spinae plane block: Plane and simple. Anaesthesia.

[59] Jiang T., Mo X., Zhan R., Zhang Y., Yu Y. (2023). Regional block techniques for pain management after video-assisted thoracoscopic surgery: A covariate-adjusted Bayesian network meta-analysis. Wideochir Inne Tech. Maloinwazyjne.

[60] Pang J., You J., Chen Y., Song C. (2023). Comparison of erector spinae plane block with paravertebral block for thoracoscopic surgery: A meta-analysis of randomized controlled trials. J. Cardiothorac. Surg..

[61] Guyatt G.H., Oxman A.D., Vist G.E., Kunz R., Falck-Ytter Y., Alonso-Coello P., Schunemann H.J., Group G.W. (2008). GRADE: An emerging consensus on rating quality of evidence and strength of recommendations. BMJ.

[62] Egger M., Davey Smith G., Schneider M., Minder C. (1997). Bias in meta-analysis detected by a simple, graphical test. BMJ.

[63] Batchelor T.J.P., Rasburn N.J., Abdelnour-Berchtold E., Brunelli A., Cerfolio R.J., Gonzalez M., Ljungqvist O., Petersen R.H., Popescu W.M., Slinger P.D. (2019). Guidelines for enhanced recovery after lung surgery: Recommendations of the Enhanced Recovery After Surgery (ERAS^®^) Society and the European Society of Thoracic Surgeons (ESTS). Eur. J. Cardiothorac. Surg..

[64] Sobhy A.A., Sharaf S.I., Kamaly A.M., Hilal A.M., Elaziz F.K.E.A. (2023). Comparative study between ultrasound-guided erector spinae plane block and thoracic paravertebral block for postoperative analgesia after video-assisted thoracic surgery: An equivalence study. Ain-Shams J. Anesthesiol..

[65] Tsui B.C.H., Fonseca A., Munshey F., McFadyen G., Caruso T.J. (2019). The erector spinae plane (ESP) block: A pooled review of 242 cases. J. Clin. Anesth..

[66] Grape S., Kirkham K.R., Frauenknecht J., Albrecht E. (2019). Intra-operative analgesia with remifentanil vs. dexmedetomidine: A systematic review and meta-analysis with trial sequential analysis. Anaesthesia.

[67] Sterne J.A.C., Savovic J., Page M.J., Elbers R.G., Blencowe N.S., Boutron I., Cates C.J., Cheng H.Y., Corbett M.S., Eldridge S.M. (2019). RoB 2: A revised tool for assessing risk of bias in randomised trials. BMJ.

[68] Hrobjartsson A., Emanuelsson F., Skou Thomsen A.S., Hilden J., Brorson S. (2014). Bias due to lack of patient blinding in clinical trials. A systematic review of trials randomizing patients to blind and nonblind sub-studies. Int. J. Epidemiol..

[69] Lonjon G., Porcher R., Ergina P., Fouet M., Boutron I. (2017). Potential Pitfalls of Reporting and Bias in Observational Studies With Propensity Score Analysis Assessing a Surgical Procedure: A Methodological Systematic Review. Ann. Surg..

