# Supplementary material for: Erector Spinae Plane Block Versus Thoracic Paravertebral Block for Postoperative Analgesia in Thoracic Surgery: A Systematic Review and Meta-Analysis of Randomized and Observational Studies
Source: J Clin Med. 2026 Feb 9;15(4):1370. doi: 10.3390/jcm15041370 (PMC12942579; doi:10.3390/jcm15041370)
Supplement: Supplementary file 1 [file jcm-15-01370-s001.zip › File S2_Search term.pdf]

Appendix A: Detailed search strategy and search terms

A.1. Search terms for Pubmed

1. thoracotomy[MeSH Terms]
2. thoracoscopy[MeSH Terms]
3. Thoracic Surgical Procedures[MeSH Terms]
4. thora*[Title/Abstract] AND surg*[Title/Abstract]
5. #1 OR #2 OR #3 OR #4
6. erector spinae plane[Title/Abstract] OR ESP block*[Title/Abstract]
7. (erector[Title/Abstract] OR paraspinal[Title/Abstract] OR thoracic neuropathic pain[Title/Abstract]) AND block*[Title/Abstract]
8. erector spinae[Title/Abstract] AND (an?esth*[Title/Abstract] OR analg*[Title/Abstract])
9. paraspinal[Title/Abstract] AND ((regional[Title/Abstract] OR local[Title/Abstract]) AND (an?esth*[Title/Abstract] OR analg*[Title/Abstract]))
10. Paraspinal Muscles[MeSH Terms] or paraspinal[Title/Abstract]

11 Nerve Block[MeSH Terms] or Anesthesia, Local[MeSH Terms]

12. #10 and #11
13. #6 or #7 or #8 or #9 or #12
14. #5 and #13

A.2. Search terms for EMBASE

1. 'thoracotomy'/exp OR 'thoracotomy'
2. 'thoracoscopy'/exp OR 'thoracoscopy'
3. 'thorax surgery'/exp OR 'thorax surgery'
4. thora* AND surg*:ti,ab
5. #1 OR #2 OR #3 OR #4
6. erector AND spinae AND plane:ti,ab
7. esp AND block*:ti,ab
8. paraspinal AND block:ti,ab
9. erector AND spinae AND an?esth*:ti,ab
10. erector AND spinae AND analg*:ti,ab
11. paraspinal AND an?esth*:ti,ab
12. paraspinal AND analg*:ti,ab
13. #6 OR #7 OR #8 OR #9 OR #10 OR #11 OR #12
14. #5 And #13

A.3. Search terms for CENTRAL

ID Search Hits

#1 MeSH descriptor: [Thoracotomy] explode all trees

#2 MeSH descriptor: [Thoracoscopy] explode all trees

#3 MeSH descriptor: [Thoracic Surgical Procedures] explode all trees

#4 (thora*):ti,ab,kw AND (surg*):ti,ab,kw (Word variations have been searched)

#5 #1 OR #2 OR #3 OR #4

#6 (erector spinae plane):ti,ab,kw OR (ESP block*):ti,ab,kw (Word variations have been searched)

#7 (erector):ti,ab,kw OR (paraspinal):ti,ab,kw OR (thoracic neuropathic pain):ti,ab,kw AND (block*):ti,ab,kw (Word variations have been searched)

#8 (erector spinae):ti,ab,kw AND (an?esth*):ti,ab,kw (Word variations have been searched)

#9 (paraspinal):ti,ab,kw AND (an?esth*):ti,ab,kw (Word variations have been searched)

#10 MeSH descriptor: [Paraspinal Muscles] explode all trees

#11 MeSH descriptor: [Nerve Block] explode all trees

#12 10 and #11

#13 #6 or #7 or #8 or #9 or #12

#14 #5 and #13

A.4. Search terms for Web of Science

Topic=(thoracotomy or thoracoscopy) and Topic=(erector spinae plane or paraspinal or esp)
