# Supplementary material for: Erector Spinae Plane Block Versus Thoracic Paravertebral Block for Postoperative Analgesia in Thoracic Surgery: A Systematic Review and Meta-Analysis of Randomized and Observational Studies
Source: J Clin Med. 2026 Feb 9;15(4):1370. doi: 10.3390/jcm15041370 (PMC12942579; doi:10.3390/jcm15041370)
Supplement: Supplementary file 1 [file jcm-15-01370-s001.zip › Table S1 _table 4_ The summary of meta_analysis and trial sequential analysis.pdf]

Table S1: The summary of meta-analysis and trial sequential analysis

		No. of studies	No. of patients	Conventional meta-analysis		Trial sequential analysis			
				RR or SMD with 95% CI	Heterogeneity (I^2; $P_{chi}{}^2$; τ; 95% PI)	Conventional test boundary	Monitoring boundary	Futility boundary	RIS
**Pain at rest-whole time**	**Total**	25	1,834	Not significant (SMD, 0.109; 95% CI, -0.037 to 0.255)	I^2 = 55.181; $P_{chi}{}^2$ <0.001; τ = 0.266; 95% PI, -0.441 to 0.659				
	**RCTs**	22	1423	Significant (SMD, 0.137; 95% CI, 0.031 to 0.243)	I^2 = 58.574; $P_{chi}{}^2$ <0.001; τ = 0.303; 95% PI, -0.494 to 0.768				
	**OSs**	3	411	Not significant (SMD, -0.058; 95% CI, -0.441 to 0.325)	I^2 = 0.0; $P_{chi}{}^2$ = 0.768; τ = 0.0				
**Pain at rest-early (0-6 h)**	**Total**	23	1718	Significant (SMD, 0.253; 95% CI, 0.034 to 0.472)	I^2 = 79.047; $P_{chi}{}^2$ <0.001; τ = 0.436; 95% PI, -0.651 to 1.157				
	**RCTs**	20	1307	Significant (SMD, 0.301; 95% CI, 0.064 to 0.538)	I^2 = 80.587; $P_{chi}{}^2$ <0.001; τ = 0.269; 95% PI, -0.262 to 0.864	Cross	Cross	Not cross	exceed RIS (1,718 vs. 1,537 patients)
	**OSs**	3	411	Not significant (SMD, -0.035; 95% CI, -0.615 to 0.546)	I^2 = 0.0; $P_{chi}{}^2$ = 0.926; τ = 0.0				
**Pain at rest-24 h**	**Total**	24	1768	Not significant (SMD, 0.077; 95% CI, -0.046 to 0.201)	I^2 = 34.137; $P_{chi}{}^2$ = 0.053; τ = 0.170; 95% PI, -0.275 to 0.429				

	**RCTs**	21	1357	Not significant (SMD, 0.106; 95% CI, -0.029 to 0.241)	I^2 = 0.0; $P_{chi}{}^2$ = 0.641; τ = 0.0	Not cross	Not cross	Cross	exceed RIS (1468 vs. 650 patients)
	**OSs**	3	411	Not significant (SMD, -0.067; 95% CI, -0.371 to 0.237)	I^2 = 88.666; $P_{chi}{}^2$ <0.001; τ = 0.544; 95% PI, -2.408 to 2.274				
**Pain at rest-48 h**	**Total (RCTs)**	12	802	Not significant (SMD, -0.154; 95% CI, -0.375 to 0.066)	I^2 = 58.375; $P_{chi}{}^2$ = 0.006; τ = 0.294; 95% PI, -0.769 to 0.461	Not cross	Not cross	Not cross	21.0% (802 vs. 3811 patients)
**Pain during cough-whole time**	**Total**	24	1789	Not significant (SMD, 0.140; 95% CI, -0.050 to 0.329)	I^2 = 71.536; $P_{chi}{}^2$ <0.001; τ = 0.383; 95% PI, -0.660 to 0.940				
	**RCTs**	21	1378	Not significant (SMD, 0.179; 95% CI, -0.025 to 0.383)	I^2 = 71.948; $P_{chi}{}^2$ <0.001; τ = 0.413; 95% PI, -0.681 to 1.039				
	**OSs**	3	411	Not significant (SMD, -0.104; 95% CI, -0.609 to 0.401)	I^2 = 68.683; $P_{chi}{}^2$ = 0.041; τ = 0.287; 95% PI, -1.336 to 1.128				
**Pain during cough-early (0-6 h)**	**Total**	21	1603	Significant (SMD, 0.280; 95% CI, 0.024 to 0.535)	I^2 = 83.755; $P_{chi}{}^2$ <0.001; τ = 0.538; 95% PI, -0.842 to 1.402				
	**RCTs**	18	1192	Significant (SMD, 0.353; 95% CI, 0.075 to 0.631)	I^2 = 84.555; $P_{chi}{}^2$ <0.001; τ = 0.595; 95% PI, -0.902 to 1.608	Cross	Not cross	Not cross	44.2% (1258 vs. 2846 patients)
	**OSs**	3	411	Not significant	I^2 = 0.0; $P_{chi}{}^2$ = 0.504; τ = 0.0				

				(SMD, -0.127; 95% CI, -0.780 to 0.527)					
**Pain at cough-24 h**	**Total**	23	1719	Not significant (SMD, 0.277; 95% CI, -0.052 to 0.606)	I^2 = 90.262; P_{chi}^2 <0.001; τ = 0.738; 95% PI, -1.250 to 1.804				
	**RCTs**	20	1308	Significant (SMD, 0.389; 95% CI, 0.036 to 0.743)	I^2 = 90.237; P_{chi}^2 <0.001; τ = 0.787; 95% PI, -1.253 to 2.031	Cross	Not cross	Not cross	66.4% (1258 vs. 1894 patients)
	**OSs**	3	411	Not significant (SMD, -0.444; 95% CI, -1.338 to 0.450)	I^2 = 90.159; P_{chi}^2 <0.001; τ = 0.593; 95% PI, -2.995 to 2.107				
**Pain during cough-48 h**	**Total (RCTs)**	12	802	Not significant (SMD, -0.017; 95% CI, -0.219 to 0.186)	I^2 = 51.048; P_{chi}^2 = 0.021; τ = 0.253; 95% PI, -0.574 to 0.540	Not cross	Not cross	Not cross	7.2% (802 vs. 11118 patients)
**Opioid consumption -whole time**	**Total**	24	1764	Significant (SMD, 0.322; 95% CI, 0.034 to 0.611)	I^2 = 87.541; P_{chi}^2 <0.001; τ = 0.646; 95% PI, -1.014 to 1.658				
	**RCTs**	21	1353	Significant (SMD, 0.358; 95% CI, 0.049 to 0.668)	I^2 = 85.006; P_{chi}^2 <0.001; τ = 0.614; 95% PI, -0.923 to 1.639				
	**OSs**	3	411	Not significant (SMD, 0.083; 95% CI, -0.714 to 0.881)	I^2 = 95.755; P_{chi}^2 <0.001; τ = 0.940; 95% PI, -3.961 to 4.127				
**Opioid consumption -24 h**	**Total**	20	1545	Significant (SMD, 0.417; 95% CI, 0.108 to 0.725)	I^2 = 87.541; P_{chi}^2 <0.001; τ = 0.646; 95% PI, -0.935 to 1.769				

	**RCTs**	17	1134	Significant (SMD, 0.438; 95% CI, 0.102 to 0.774)	I^2 = 86.704; P_{chi}^2 <0.001; τ = 0.650; 95% PI, -0.940 to 1.816	Cross	Not cross	Not cross	exceed RIS (1,718 vs. 732 patients).
	**OSs**	3	411	Not significant (SMD, 0.303; 95% CI, -0.474 to 1.080)	I^2 = 92.297; P_{chi}^2 <0.001; τ = 0.672; 95% PI, -2.588 to 3.194				
**Opioid consumption -48 h**	**Total (RCTs)**	11	715	Not significant (SMD, 0.241; 95% CI, -0.112 to 0.595)	I^2 = 81.545; P_{chi}^2 <0.001; τ = 0.536; 95% PI, -0.953 to 1.435	Not cross	Not cross	Not cross	7.2% (802 vs. 11118 patients)
**PONV**	**Total**	17	1,132	Not significant (RR, 0.909; 95% CI, 0.704 to 1.174)	I^2 = 2.372; P_{chi}^2 = 0.426; τ = 0.085; 95% PI, 0.320 to 2.582				
	**RCTs**	15	1,005	Not significant (RR, 0.976; 95% CI, 0.730 to 1.305)	I^2 = 2.012; P_{chi}^2 = 0.429; τ = 0.084; 95% PI, 0.316 to 3.011	Not cross	Not cross	Not cross	47.1% (1005 vs. 2135 patients)
	**OSs**	2	127	Not significant (RR, 0.714; 95% CI, 0.417 to 1.223)	I^2 = 0.0; P_{chi}^2 = 0.320; τ = 0.0				
**Hypotension**	**Total**	10	829	Not significant (RR, 0.680; 95% CI, 0.359 to 1.291)	I^2 = 41.119; P_{chi}^2 = 0.083; τ = 0.510; 95% PI, 0.198 to 2.337				
	**RCTs**	9	579	Not significant (RR, 0.574; 95% CI, 0.279 to 1.184)	I^2 = 40.314; P_{chi}^2 = 0.099; τ = 0.636; 95% PI, 0.247 to 1.331	Not cross	Not cross	Not cross	13.4% (579 vs. 4317 patients)
	**OS**	1	250	Not significant					

	(RR, 1.257; 95% CI, 0.703 to 2.249)	

CI, confidence interval; I^2, Higgins' inconsistency index; No., number; OS, observational study; PI, prediction interval; $Pchi^2$, p value for Cochran's Q test; PONV, postoperative nausea and vomiting; RCT, randomized controlled trial; RIS, required information size; RR, relative risk; SMD, standardized mean difference; τ, between-study standard deviation.
