# Supplementary material for: Erector Spinae Plane Block Versus Thoracic Paravertebral Block for Postoperative Analgesia in Thoracic Surgery: A Systematic Review and Meta-Analysis of Randomized and Observational Studies
Source: J Clin Med. 2026 Feb 9;15(4):1370. doi: 10.3390/jcm15041370 (PMC12942579; doi:10.3390/jcm15041370)
Supplement: Supplementary file 1 [file jcm-15-01370-s001.zip › Table S2 _Table 5. Certainty of evidence.pdf]

Table S2: Certainty of evidence

		No. of studies	No. of patients	Effect Estimate	Quality					
					ROB	Inconsistency	Indirectness	Imprecision	Publication Bias	
**Pain at rest-whole time**	**Total**	25	1,834	SMD, 0.109; 95% CI, -0.037 to 0.255	Not serious	Serious	Not serious	Not serious	Not serious	⊕⊕⊕◯ MODERATE
	**RCTs**	22	1423	SMD, 0.137; 95% CI, 0.031 to 0.243	Not serious	Serious	Not serious	Not serious	Not serious	⊕⊕⊕◯ MODERATE
	**OSs**	3	411	SMD, -0.058; 95% CI, -0.441 to 0.325	Serious	Not serious	Not serious	Serious	Not detected	⊕◯◯◯ VERY LOW
**Pain at rest-early (0-6 h)**	**Total**	23	1718	SMD, 0.253; 95% CI, 0.034 to 0.472	Not serious	Serious	Not serious	Not serious	Not serious	⊕⊕⊕◯ MODERATE
	**RCTs**	20	1307	SMD, 0.301; 95% CI, 0.064 to 0.538	Not serious	Serious	Not serious	Not serious	Not serious	⊕⊕⊕◯ MODERATE
	**OSs**	3	411	SMD, -0.035; 95% CI, -0.615 to 0.546	Serious	Not serious	Not serious	Serious	Not detected	⊕◯◯◯ VERY LOW
**Pain at rest-24 h**	**Total**	24	1768	SMD, 0.077; 95% CI, -0.046 to 0.201	Not serious	Not serious	Not serious	Not serious	Not serious	⊕⊕⊕⊕ HIGH

	**RCTs**	21	1357	SMD, 0.106; 95% CI, -0.029 to 0.241	Not serious	Not serious	Not serious	Not serious	Not serious	⊕⊕⊕⊕ HIGH
	**OSs**	3	411	SMD, -0.067; 95% CI, -0.371 to 0.237	Serious	Serious	Not serious	Serious	Not detected	⊕◯◯◯ VERY LOW
**Pain at rest-48 h**	**Total (RCTs)**	12	802	SMD, -0.154; 95% CI, -0.375 to 0.066	Not serious	Serious	Not serious	Not serious	Not serious	⊕⊕⊕◯ MODERATE
**Pain during cough-whole time**	**Total**	24	1789	SMD, 0.140; 95% CI, -0.050 to 0.329	Not serious	Serious	Not serious	Not serious	Not serious	⊕⊕⊕◯ MODERATE
	**RCTs**	21	1378	SMD, 0.179; 95% CI, -0.025 to 0.383	Not serious	Serious	Not serious	Not serious	Not serious	⊕⊕⊕◯ MODERATE
	**OSs**	3	411	SMD, -0.104; 95% CI, -0.609 to 0.401	Serious	Serious	Not serious	Serious	Not detected	⊕◯◯◯ VERY LOW
**Pain during cough-early (0-6 h)**	**Total**	21	1603	SMD, 0.280; 95% CI, 0.024 to 0.535	Not serious	Serious	Not serious	Not serious	Not serious	⊕⊕⊕◯ MODERATE
	**RCTs**	18	1192	SMD, 0.353; 95% CI, 0.075 to 0.631	Not serious	Serious	Not serious	Not serious	Not serious	⊕⊕⊕◯ MODERATE
	**OSs**	3	411	SMD, -0.127; 95% CI, -0.780 to 0.527	Serious	Not serious	Not serious	Serious	Not detected	⊕◯◯◯ VERY LOW

**Pain during cough-24 h**	**Total**	23	1719	SMD, 0.277; 95% CI, -0.052 to 0.606	Not serious	Serious	Not serious	Not serious	Not serious	⊕⊕⊕◯ MODERATE
	**RCTs**	20	1308	SMD, 0.389; 95% CI, 0.036 to 0.743	Not serious	Serious	Not serious	Not serious	Not serious	⊕⊕⊕◯ MODERATE
	**OSs**	3	411	SMD, -0.444; 95% CI, -1.338 to 0.450	Serious	Serious	Not serious	Serious	Not detected	⊕◯◯◯ VERY LOW
**Pain during cough-48 h**	**Total (RCTs)**	12	802	SMD, -0.017; 95% CI, -0.219 to 0.186	Not serious	Serious	Not serious	Not serious	Not serious	⊕⊕⊕◯ MODERATE
**Opioid consumption-whole time**	**Total**	24	1764	SMD, 0.322; 95% CI, 0.034 to 0.611	Not serious	Serious	Not serious	Not serious	Not serious	⊕⊕⊕◯ MODERATE
	**RCTs**	21	1353	SMD, 0.358; 95% CI, 0.049 to 0.668	Not serious	Serious	Not serious	Not serious	Not serious	⊕⊕⊕◯ MODERATE
	**OSs**	3	411	SMD, 0.083; 95% CI, -0.714 to 0.881	Serious	Serious	Not serious	Serious	Not detected	⊕◯◯◯ VERY LOW
**Opioid consumption-24 h**	**Total**	20	1545	SMD, 0.417; 95% CI, 0.108 to 0.725	Not serious	Serious	Not serious	Not serious	Not serious	⊕⊕⊕◯ MODERATE
	**RCTs**	17	1134	SMD, 0.438; 95% CI, 0.102 to 0.774	Not serious	Serious	Not serious	Not serious	Not serious	⊕⊕⊕◯ MODERATE

	**OSs**	3	411	SMD, 0.303; 95% CI, -0.474 to 1.080	Serious	Serious	Not serious	Serious	Not detected	⊕◯◯◯ VERY LOW
**Opioid consumption-48 h**	**Total (RCTs)**	11	715	SMD, 0.241; 95% CI, -0.112 to 0.595	Not serious	Serious	Not serious	Not serious	Not serious	⊕⊕⊕◯ MODERATE
**PONV**	**Total**	17	1,132	RR, 0.909; 95% CI, 0.704 to 1.174	Not serious	Not serious	Not serious	Not serious	Not serious	⊕⊕⊕⊕ HIGH
	**RCTs**	15	1,005	RR, 0.976; 95% CI, 0.730 to 1.305	Not serious	Not serious	Not serious	Not serious	Not serious	⊕⊕⊕⊕ HIGH
	**OSs**	2	127	RR, 0.714; 95% CI, 0.417 to 1.223	Serious	Not serious	Not serious	Serious	Not detected	⊕◯◯◯ VERY LOW
**Hypotension**	**Total**	10	829	RR, 0.680; 95% CI, 0.359 to 1.291	Not serious	Not serious	Not serious	Not serious	Not serious	⊕⊕⊕⊕ HIGH
	**RCTs**	9	579	RR, 0.574; 95% CI, 0.279 to 1.184	Not serious	Not serious	Not serious	Not serious	Not detected	⊕⊕⊕⊕ HIGH
	**OSs**	1	250	RR, 1.257; 95% CI, 0.703 to 2.249	Serious	Not serious	Not serious	Serious	Not detected	⊕◯◯◯ VERY LOW

CI, confidence interval; No., number; OS, observational study; PONV, postoperative nausea and vomiting; RCT, randomized controlled trial; ROB, risk of bias; RR, relative risk; SMD, standardized mean difference.
