# Supplementary material for: Erector Spinae Plane Block Versus Thoracic Paravertebral Block for Postoperative Analgesia in Thoracic Surgery: A Systematic Review and Meta-Analysis of Randomized and Observational Studies
Source: J Clin Med. 2026 Feb 9;15(4):1370. doi: 10.3390/jcm15041370 (PMC12942579; doi:10.3390/jcm15041370)
Supplement: Supplementary file 1 [file jcm-15-01370-s001.zip › Doc S1_Figure S13_publication bias assessment.pdf]

Figure S13: Funnel plots for publication bias assessment. No substantial asymmetry was observed, and Egger's regression test and Begg's rank correlation test indicated no evidence of publication bias.

Figure S13a: Pain at rest across all postoperative time points (whole time).

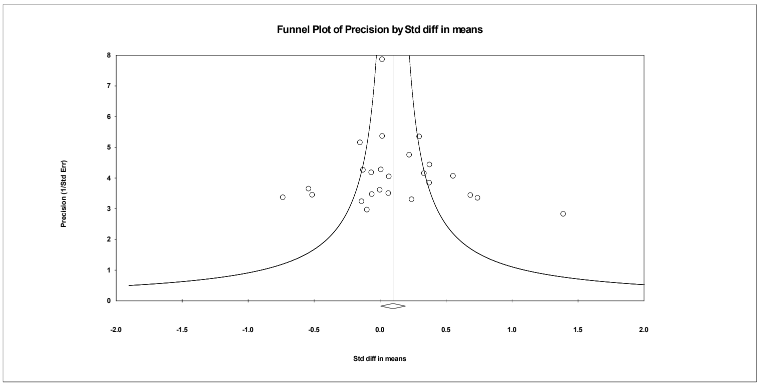

Figure S13b: Pain at rest at early postoperative period (0–6 h).

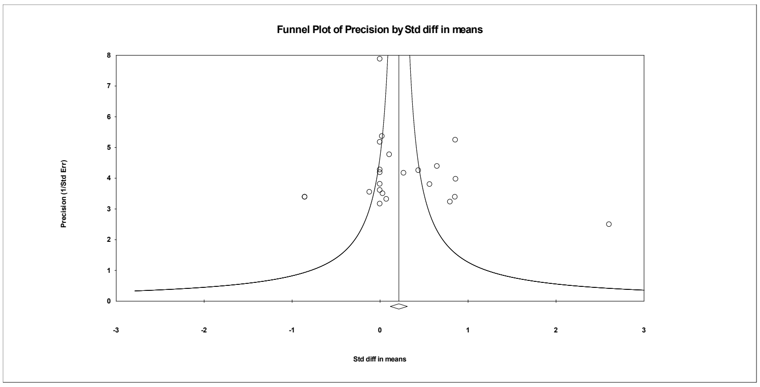

Figure S13c: Pain at rest at 24 h postoperatively.

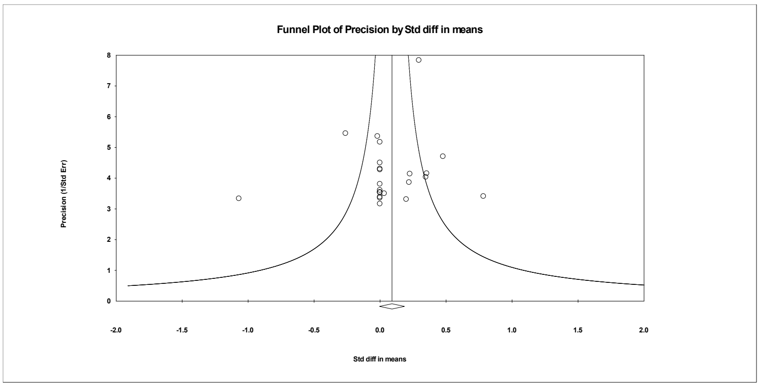

Figure S13d: Pain at rest at 48 h postoperatively.

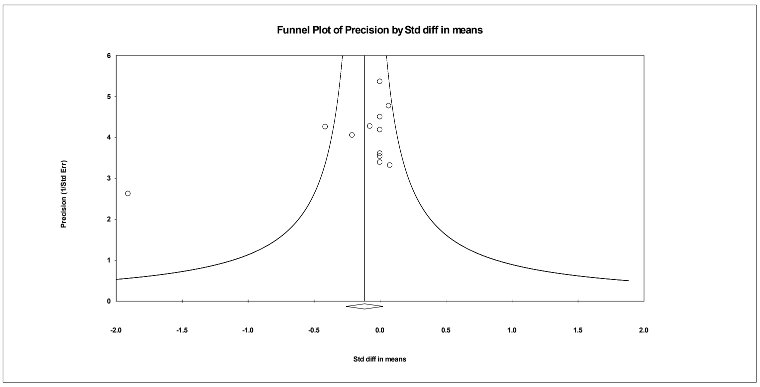

Figure S13e: Pain during coughing across all postoperative time points (whole time).

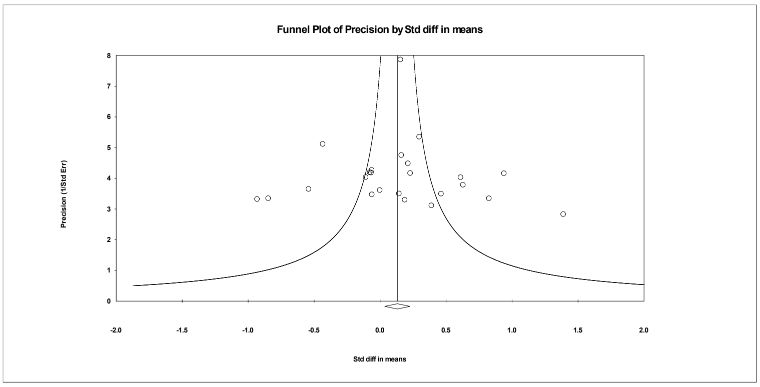

Figure S13f: Pain during coughing early postoperative period (0–6 h).

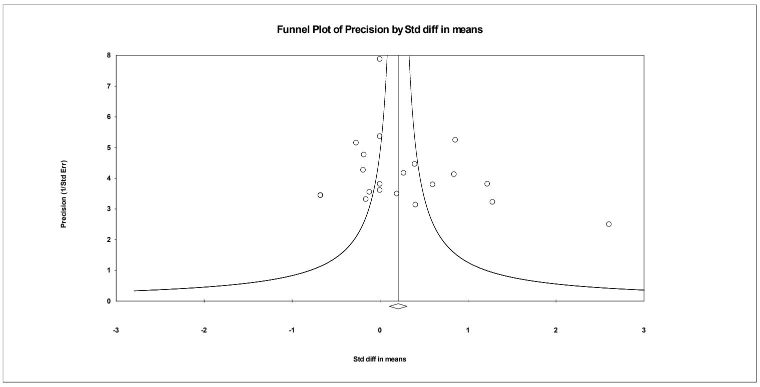

Figure S13g: Pain during coughing at 24 h postoperatively.

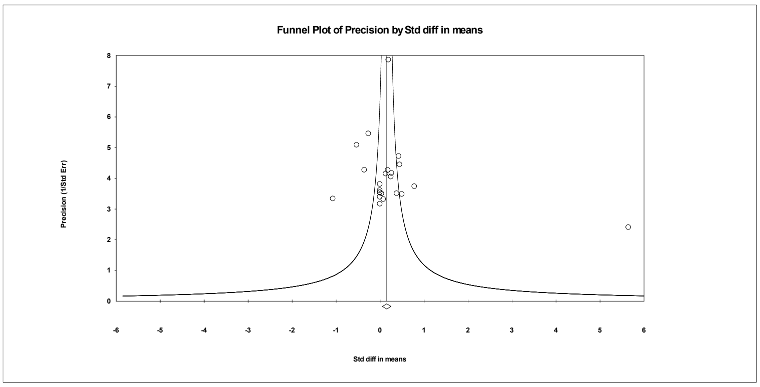

Figure S13h: Pain during coughing at 48 h postoperatively.

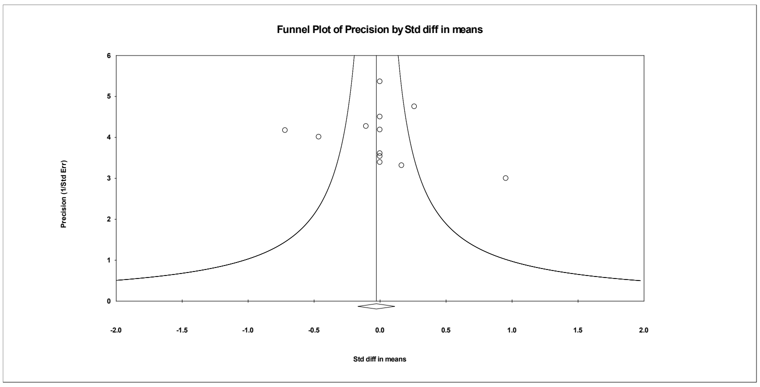

Figure S13i: Cumulative opioid consumption across all postoperative time points (whole time).

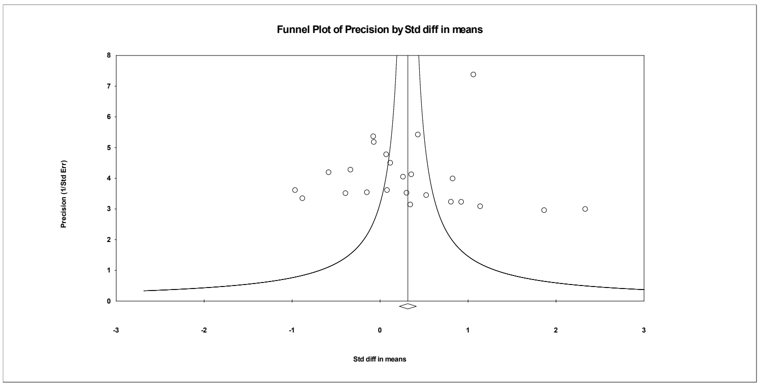

Figure S13j: Cumulative opioid consumption at 24 h postoperatively.

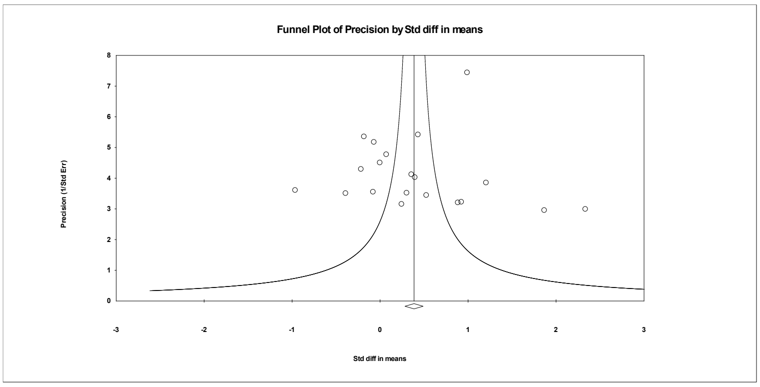

Figure S13k: Cumulative opioid consumption at 48 h postoperatively.

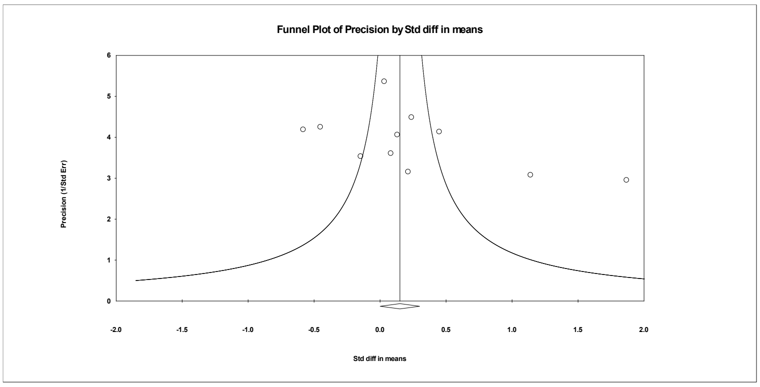

Figure S13l: Postoperative nausea and vomiting (PONV).

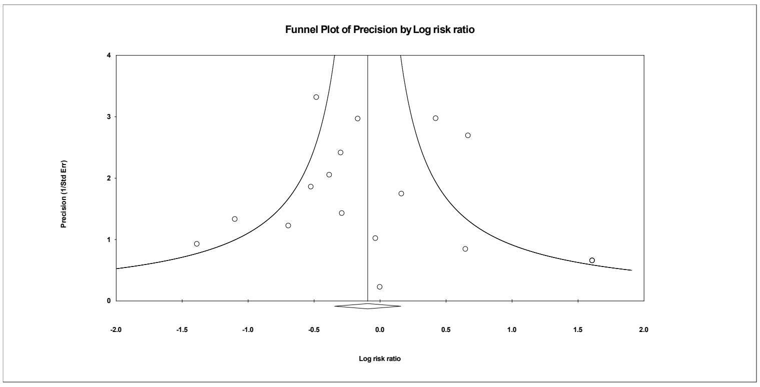

Figure S13m: Hypotension.

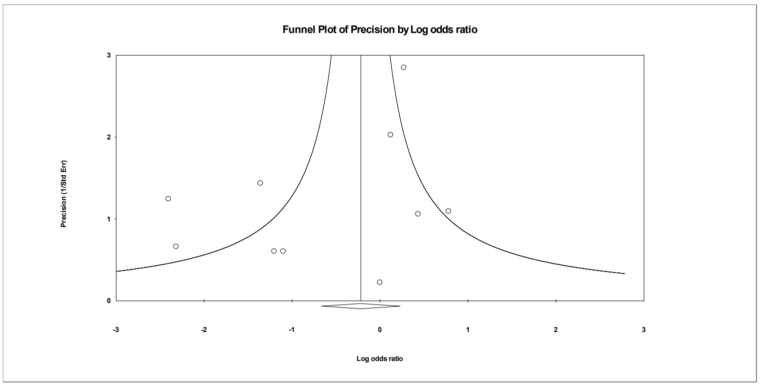

Publication bias was assessed for 13 outcomes that included at least 10 studies. Visual inspection of the funnel plots revealed a relatively symmetric distribution of studies around the pooled effect estimate for all outcomes (**Figure S13a–m**).

Pain at rest

- **Whole time:** Egger's regression test showed no evidence of publication bias (bias coefficient = 0.693, 95% CI −1.862 to 3.249, $p = 0.580$), and Begg's rank correlation test was also not significant (Kendall's

tau = 0.057, p = 0.691) (**Figure S13a**).

- **Early:** Egger's regression test indicated no evidence of publication bias (bias coefficient = 1.745, 95% CI −2.008 to 1.805, p = 0.345), and Begg's rank correlation test was not significant (Kendall's tau = 0.123, p = 0.413) (**Figure S13b**).
- **24 h:** Egger's regression test showed no evidence of publication bias (bias coefficient = −1.000, 95% CI −3.207 to 1.207, p = 0.358), and Begg's rank correlation test was not significant (Kendall's tau = −0.183, p = 0.215) (**Figure S13c**).
- **48 h:** Egger's regression test showed no statistically significant evidence of publication bias (bias coefficient = −4.817, 95% CI −9.764 to 2.220, p = 0.055), and Begg's rank correlation test was not significant (Kendall's tau = −0.348, p = 0.115) (**Figure S13d**).

Pain during cough

- **Whole time:** Egger's regression test demonstrated no evidence of publication bias (bias coefficient = 0.448, 95% CI −2.931 to 1.629, p = 0.786), and Begg's rank correlation test was not significant (Kendall's tau = 0.072, p = 0.637) (**Figure S13e**).
- **Early:** Egger's regression test showed no evidence of publication bias (bias coefficient = 2.737, 95% CI −1.538 to 7.011, p = 0.196), and Begg's rank correlation test was not significant (Kendall's tau = 0.124, p = 0.432) (**Figure S13f**).
- **24 h:** Egger's regression test demonstrated no evidence of publication bias (bias coefficient = 3.957, 95% CI −1.428 to 9.341, p = 0.141), and Begg's rank correlation test was not significant (Kendall's tau = 0.067, p = 0.653) (**Figure S13g**).
- **48 h:** Egger's regression test showed no evidence of publication bias (bias coefficient = 2.302, 95% CI −3.524 to 8.129, p = 0.399), and Begg's rank correlation test was not significant (Kendall's tau = −0.015, p = 0.945) (**Figure S13h**).

Opioid consumption

- **Whole time:** Egger's regression test indicated no evidence of publication bias (bias coefficient = −0.325, 95% CI −5.372 to 4.721, p = 0.895), and Begg's rank correlation test was not significant (Kendall's tau = 0.264, p = 0.070) (**Figure S13i**).
- **24 h:** Egger's regression test showed no evidence of publication bias (bias coefficient = 0.607, 95% CI −4.742 to 5.956, p = 0.814), and Begg's rank correlation test was not significant (Kendall's tau = 0.258, p = 0.112) (**Figure S13j**).
- **48 h:** Egger's regression test demonstrated no evidence of publication bias (bias coefficient = 7.180, 95% CI −0.858 to 15.218, p = 0.074), and Begg's rank correlation test was not significant (Kendall's tau = 0.364, p = 0.119) (**Figure S13k**).

Postoperative nausea and vomiting (PONV)

Egger’s regression test showed no evidence of publication bias (bias coefficient = 0.017, 95% CI −1.111 to 1.144, p = 0.975), and Begg’s rank correlation test was not significant (Kendall’s tau = 0.096, p = 0.592) (**Figure S13l**).

Hypotension

Egger’s regression test demonstrated no evidence of publication bias (bias coefficient = −1.047, 95% CI −2.844 to 0.751, p = 0.216), and Begg’s rank correlation test was not significant (Kendall’s tau = −0.133, p = 0.592) (**Figure S13m**).
