# Supplementary figures and images for: Erector Spinae Plane Block Versus Thoracic Paravertebral Block for Postoperative Analgesia in Thoracic Surgery: A Systematic Review and Meta-Analysis of Randomized and Observational Studies
Source: J Clin Med. 2026 Feb 9;15(4):1370. doi: 10.3390/jcm15041370 (PMC12942579; doi:10.3390/jcm15041370)

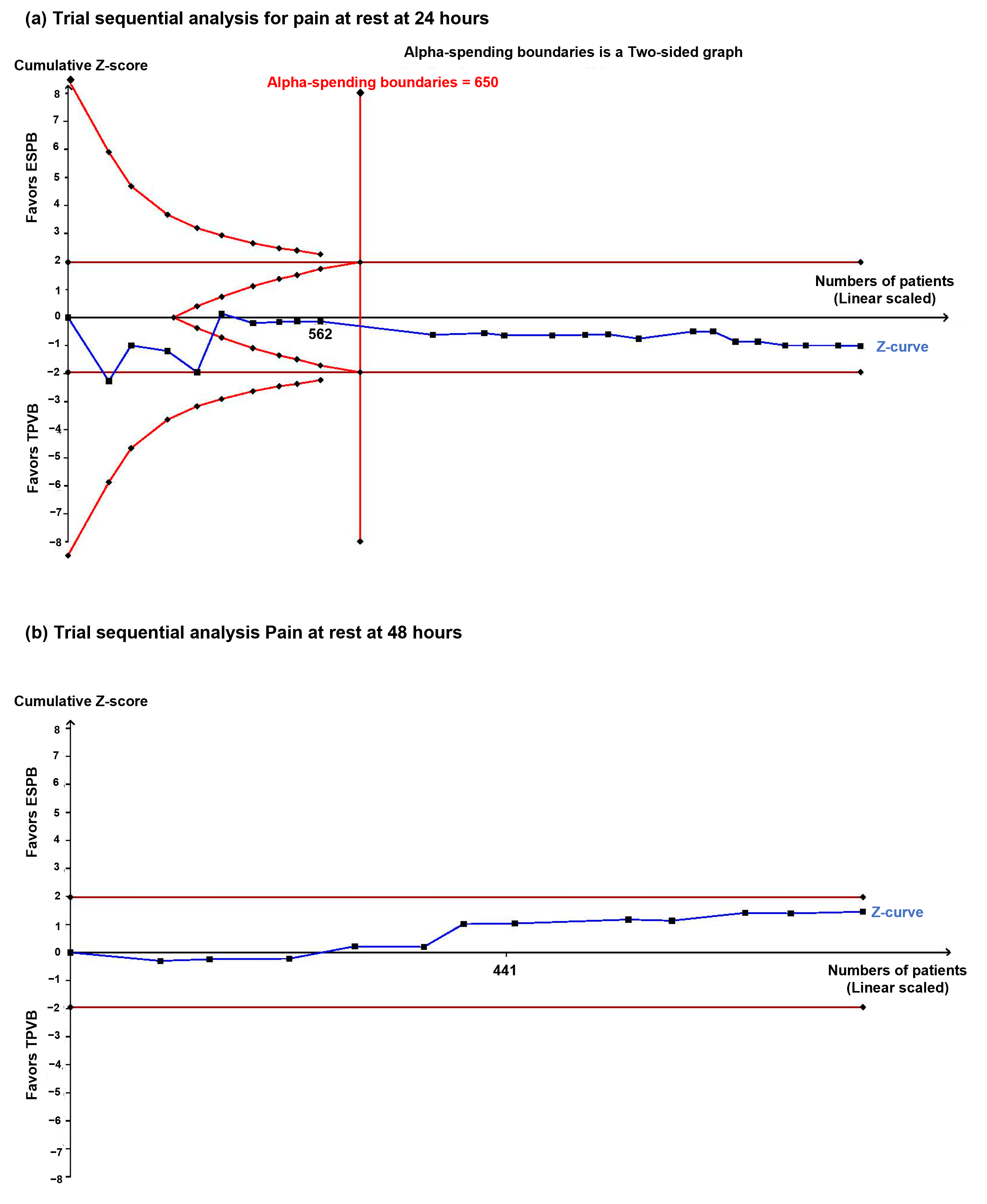

Supplement: Supplementary file 1 [file jcm-15-01370-s001.zip › Figure S3 meta rest TSA_revision.png]

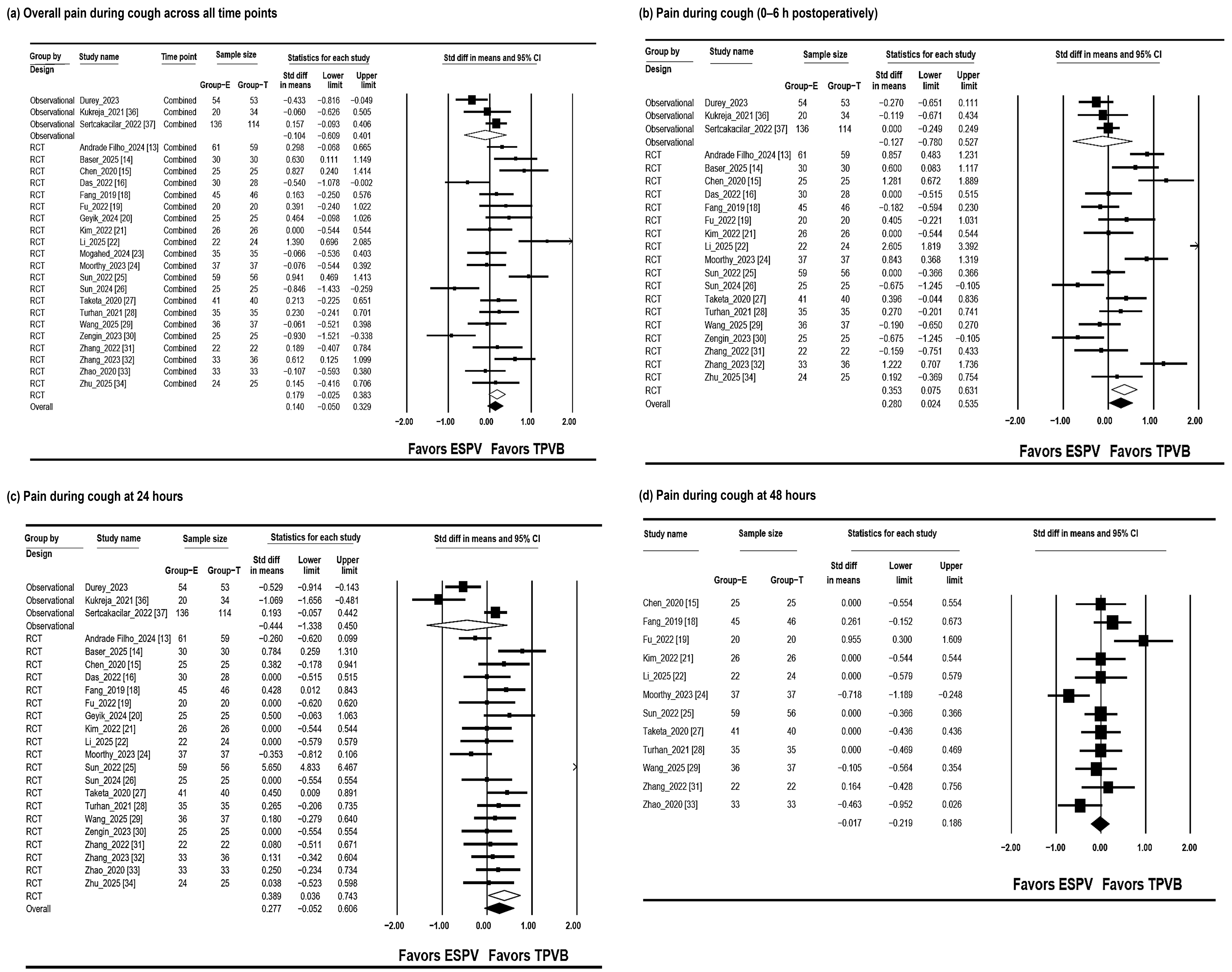

Supplement: Supplementary file 1 [file jcm-15-01370-s001.zip › Figure S4 meta cough_revision (2nd).png]

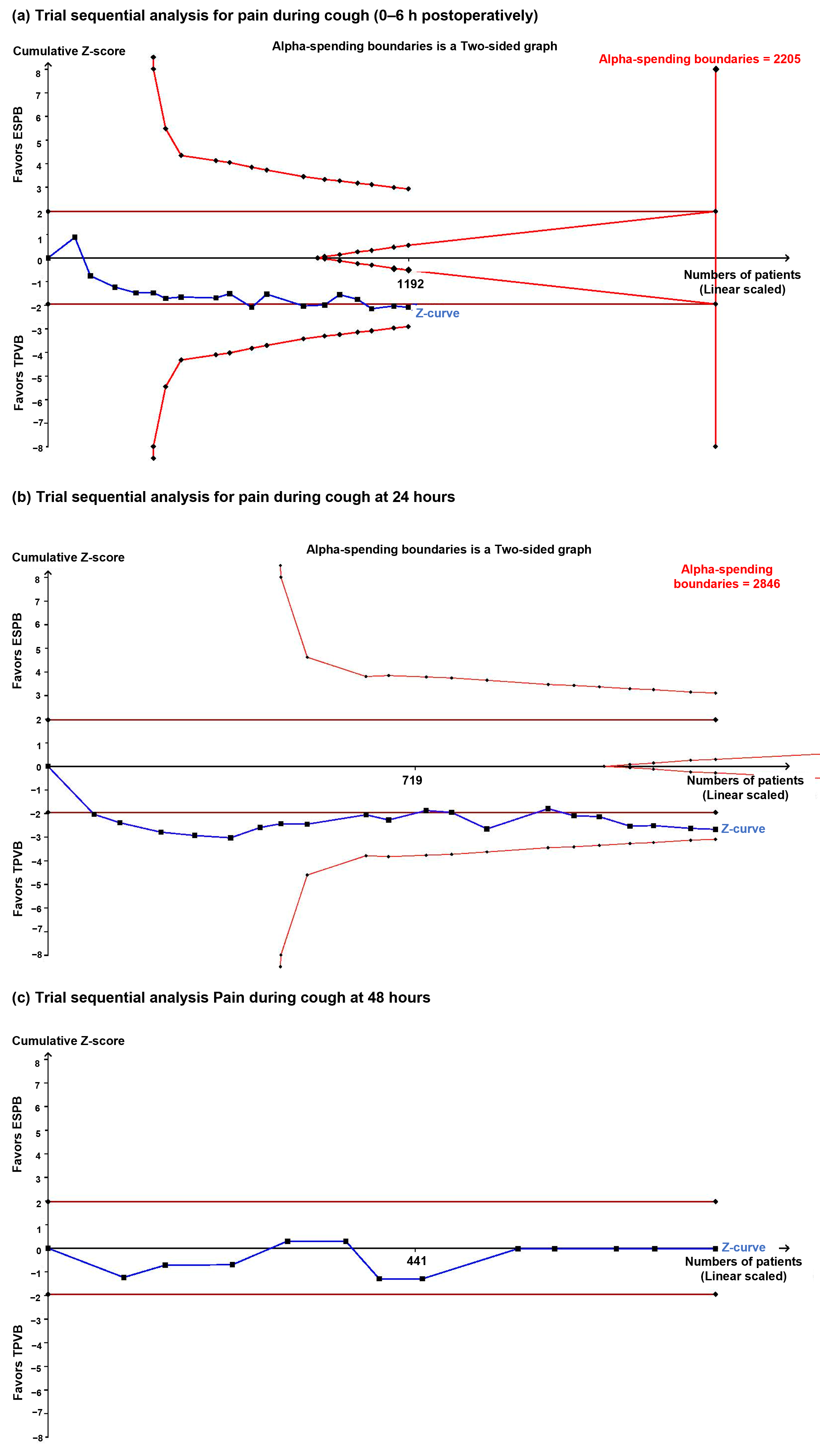

Supplement: Supplementary file 1 [file jcm-15-01370-s001.zip › Figure S6 meta cough TSA_revision.png]

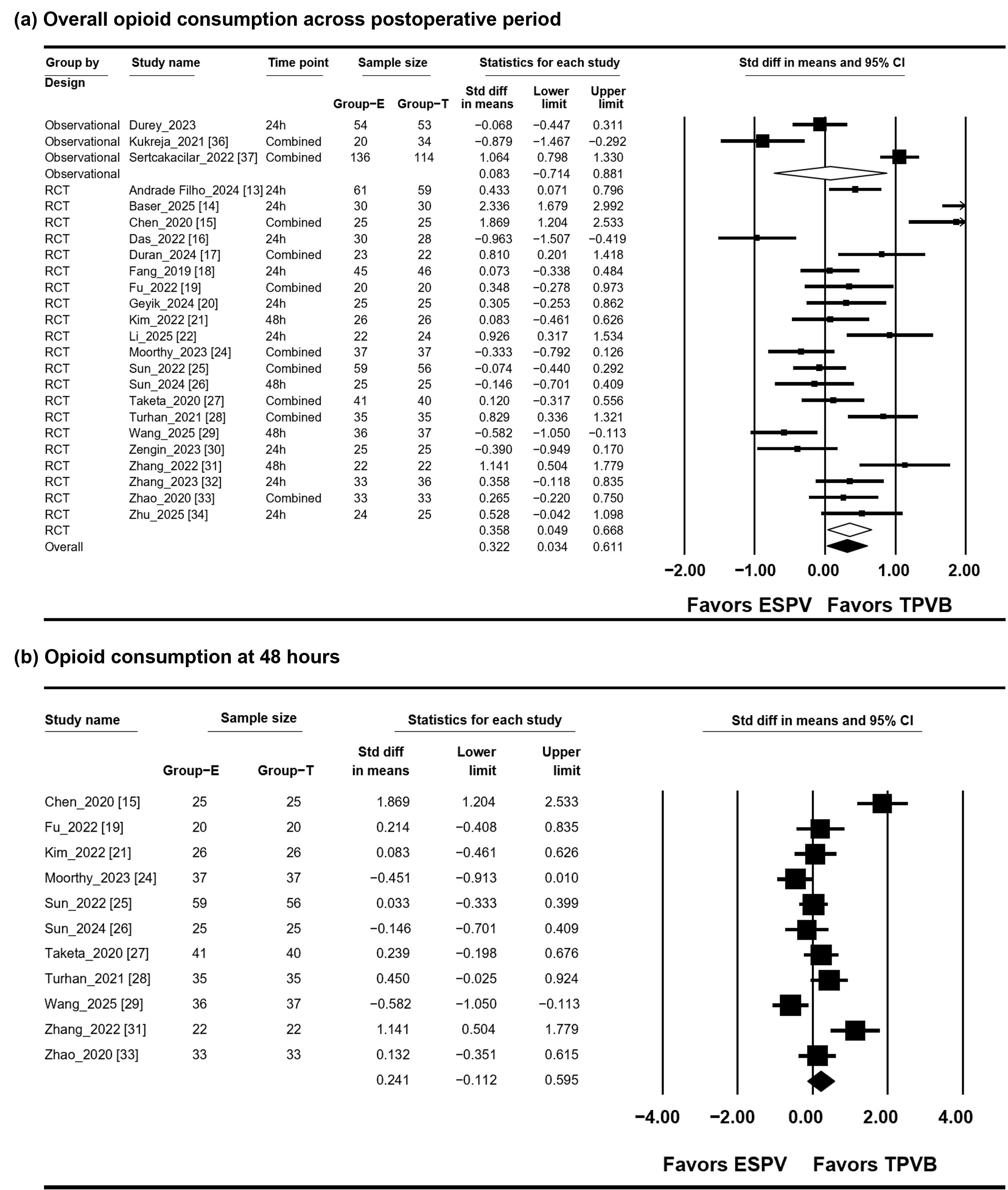

Supplement: Supplementary file 1 [file jcm-15-01370-s001.zip › Figure S7 meta Opioid_revision.png]

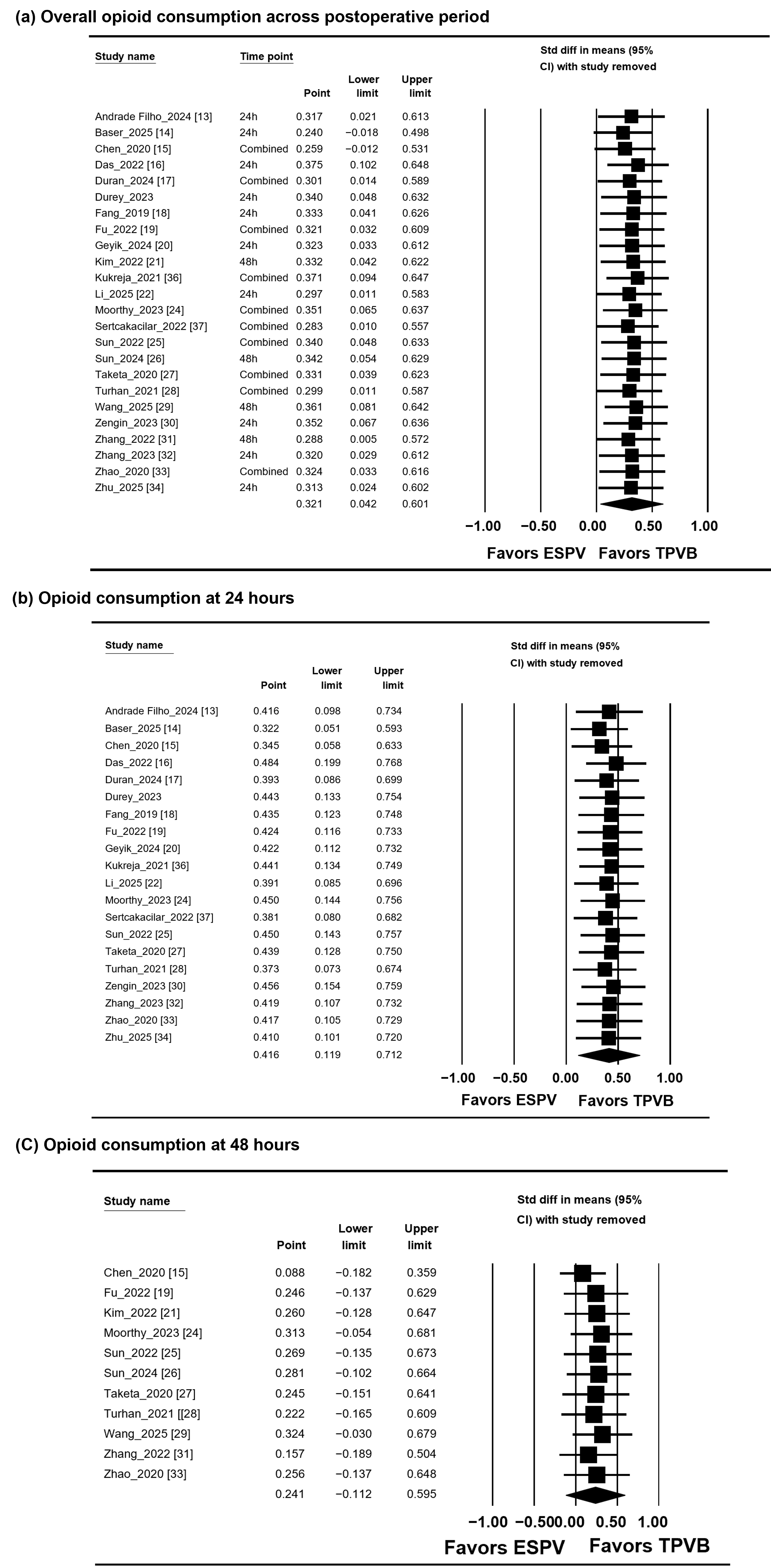

Supplement: Supplementary file 1 [file jcm-15-01370-s001.zip › Figure S8 meta Opioid sensitivity_revision.png]

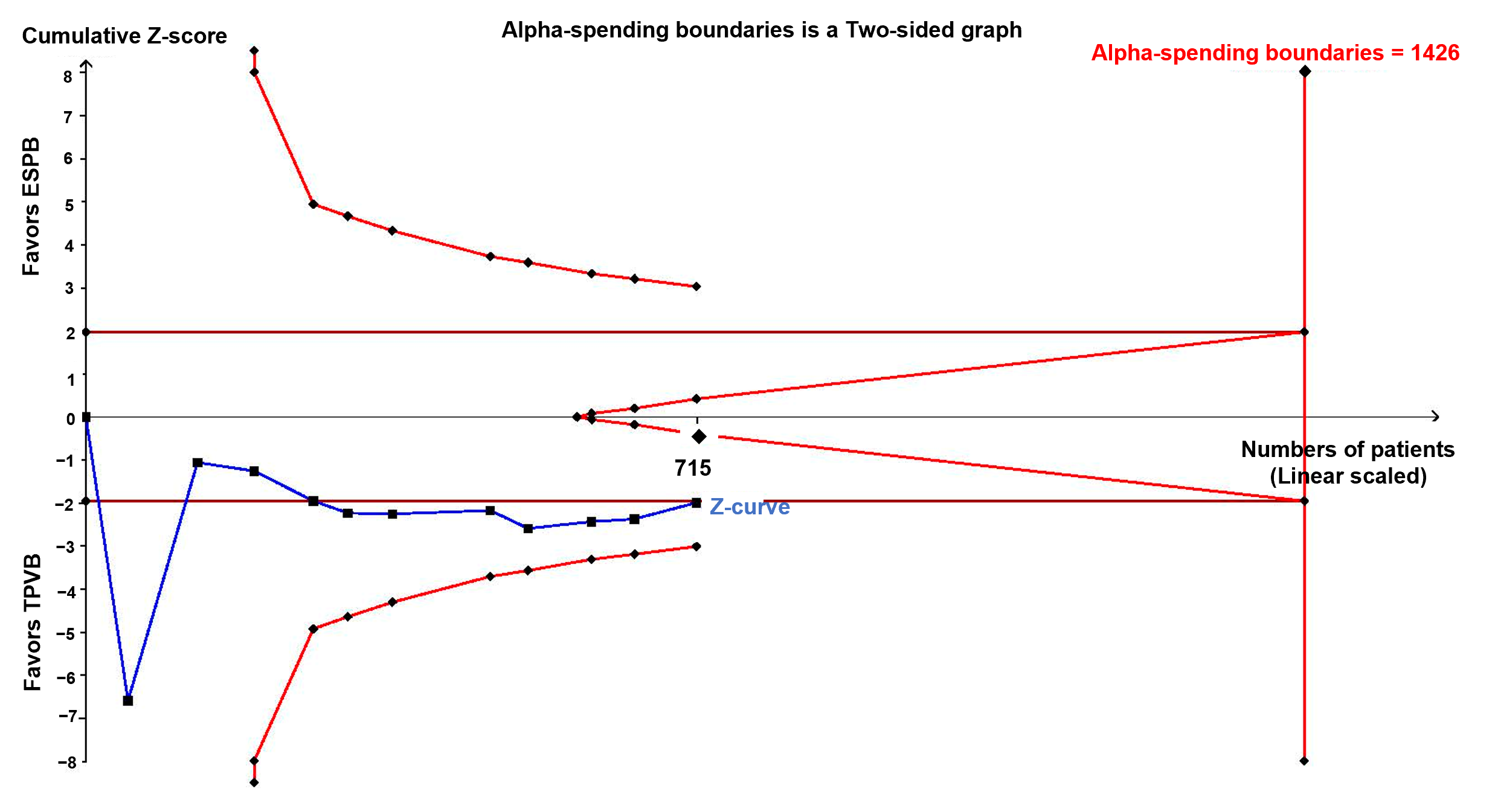

Supplement: Supplementary file 1 [file jcm-15-01370-s001.zip › Figure S9 meta Opioid TSA_revision.png]

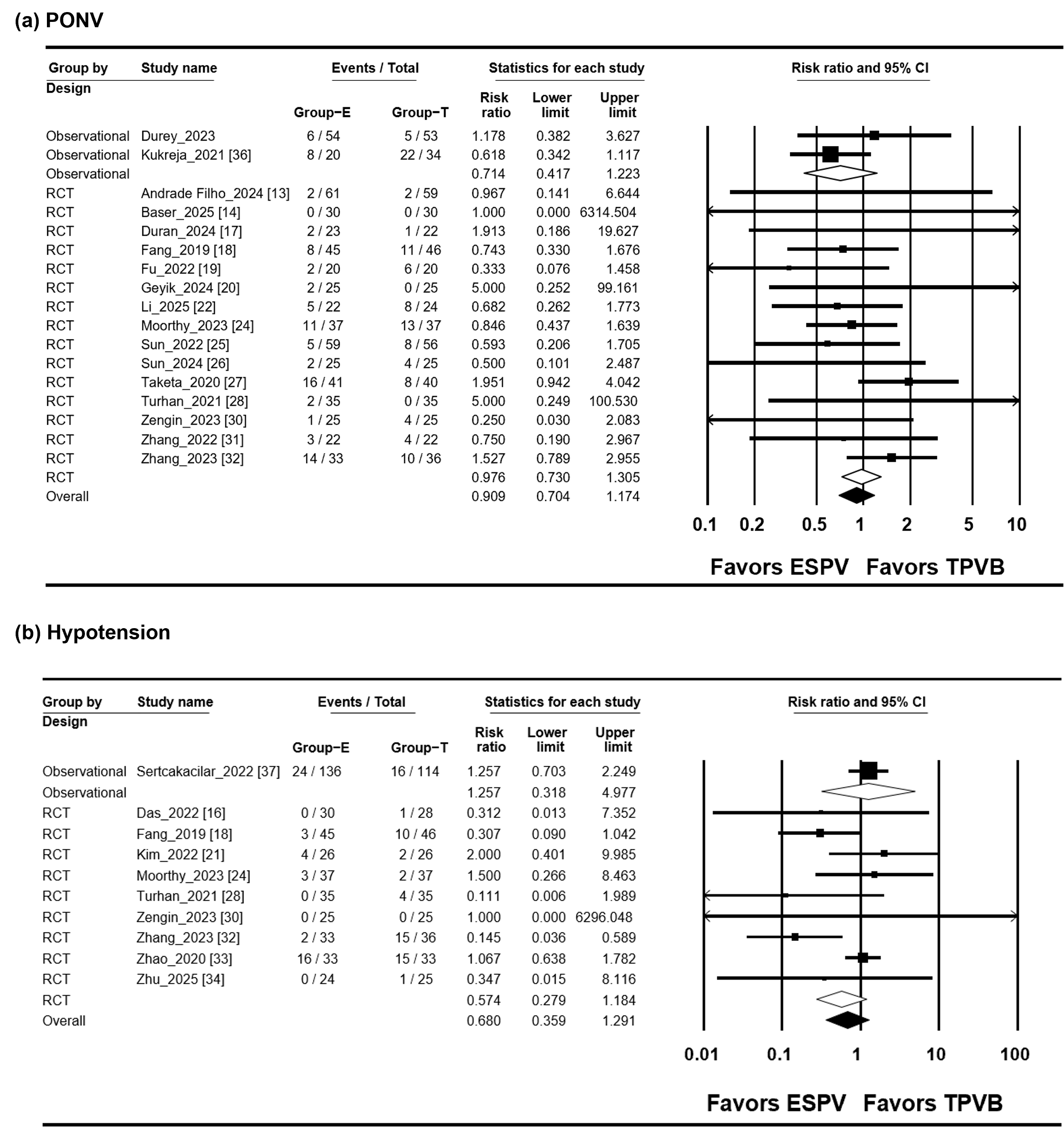

Supplement: Supplementary file 1 [file jcm-15-01370-s001.zip › Figure S10 meta PONV Hypotension_revision.png]

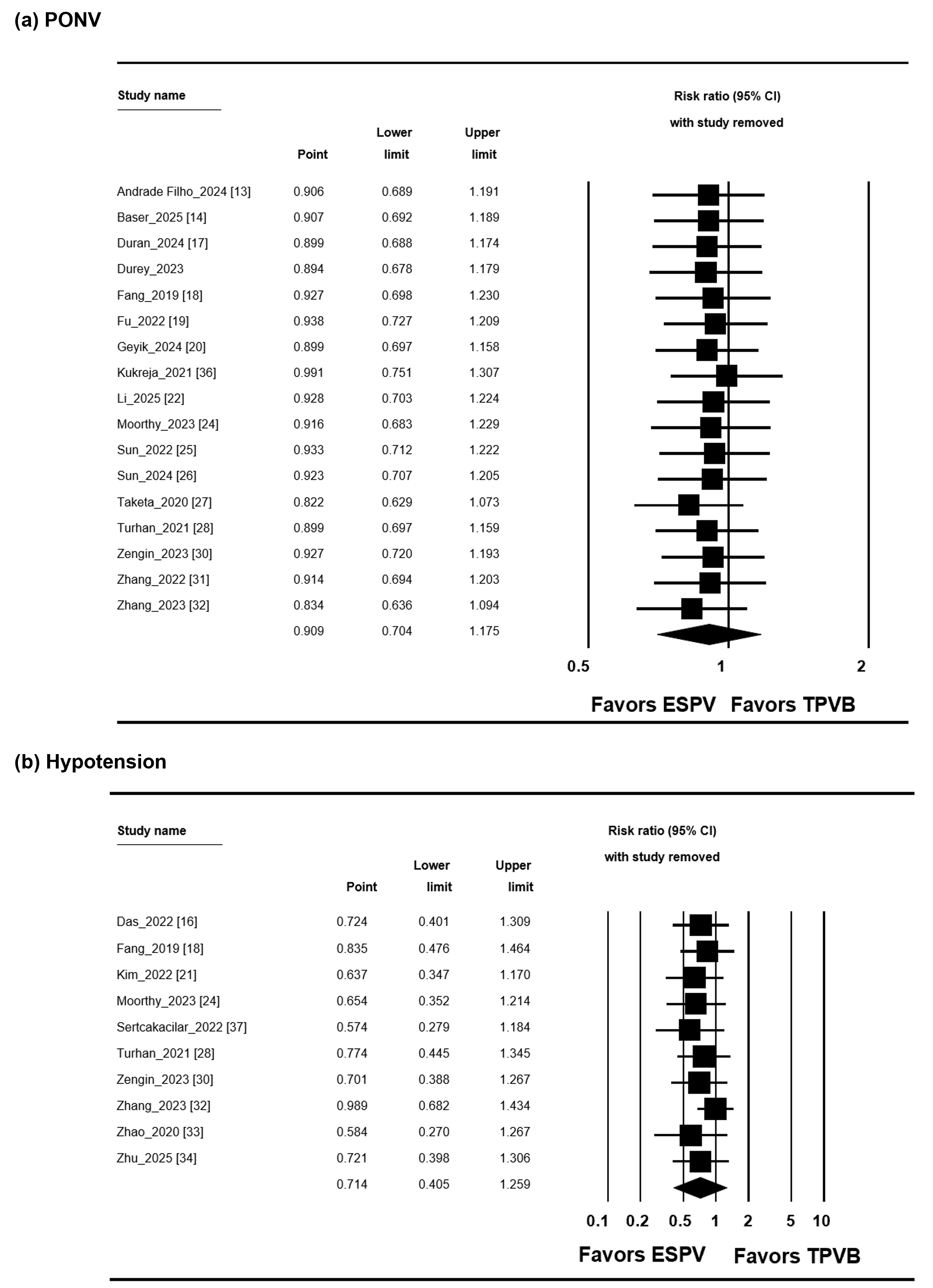

Supplement: Supplementary file 1 [file jcm-15-01370-s001.zip › Figure S11 meta PONV Hypotension sensitivity_revision.png]

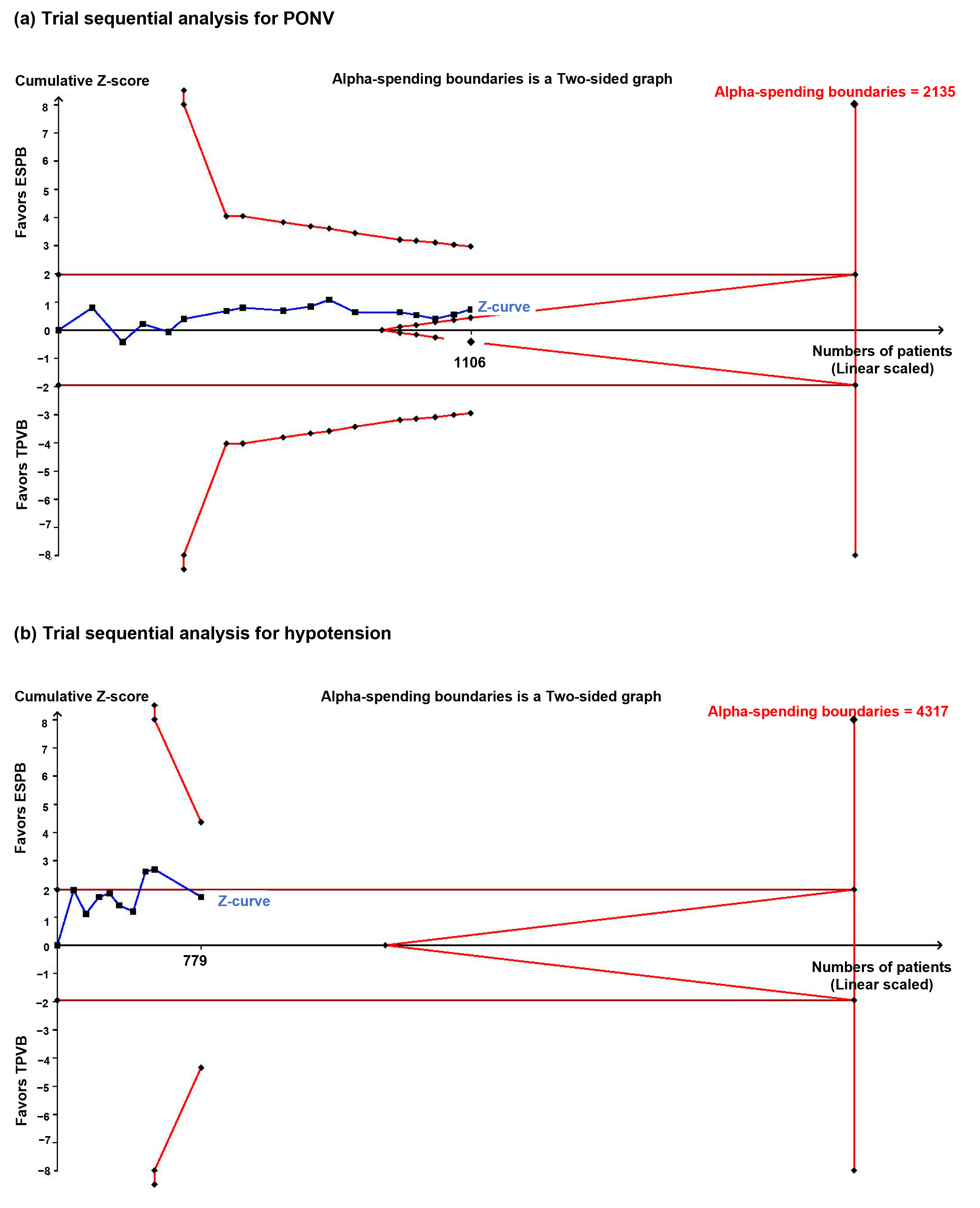

Supplement: Supplementary file 1 [file jcm-15-01370-s001.zip › Figure S12 meta PONV HypotensionTSA_revision.png]

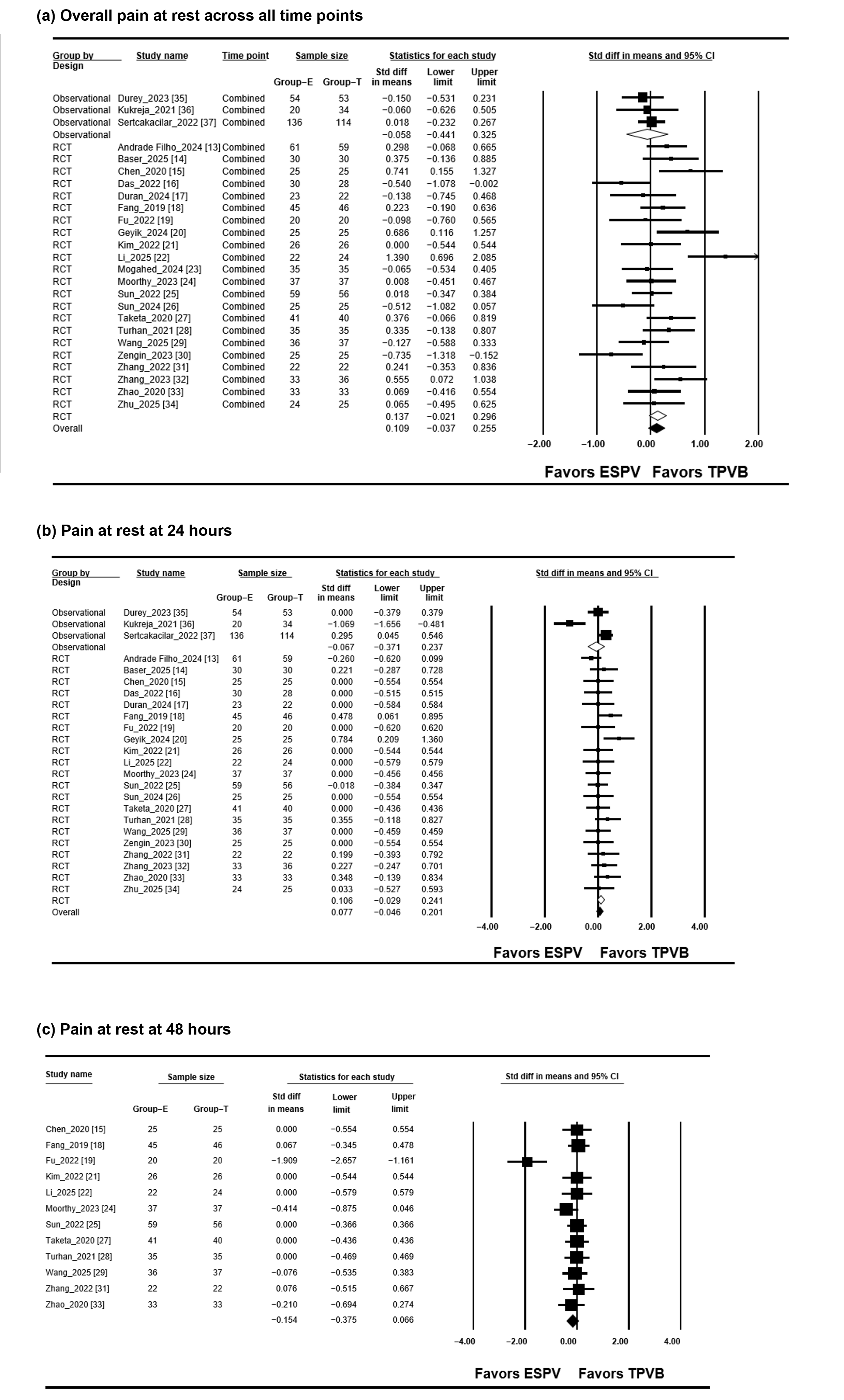

Supplement: Supplementary file 1 [file jcm-15-01370-s001.zip › Figure S1 meta rest_revision.png]
